# Synergistic Anticancer Strategy Targeting ECM Stiffness: Integration of Matrix Softening and Mechanical Signal Transduction Blockade in Primary Liver Cancers

**DOI:** 10.1002/advs.202403040

**Published:** 2024-12-20

**Authors:** Zefeng Shen, Liye Tao, Yali Wang, Yiwei Zhu, Haoyu Pan, Yijun Li, Shi Jiang, Junhao Zheng, Jingwei Cai, Yang Liu, Kainan Lin, Shihao Li, Yifan Tong, Liqing Shangguan, Junjie Xu, Xiao Liang

**Affiliations:** ^1^ Zhejiang Key Laboratory of Multi‐omics Precision Diagnosis and Treatment of Liver Diseases Department of General Surgery Sir Run Run Shaw Hospital Zhejiang University School of Medicine Hangzhou Zhejiang 310016 China; ^2^ Zhejiang Minimal Invasive Diagnosis and Treatment Technology Research Center of Severe Hepatobiliary Disease Zhejiang Research and Development Engineering Laboratory of Minimally Invasive Technology and Equipment Hangzhou Zhejiang 310016 China; ^3^ Zhejiang University Cancer Center Hangzhou Zhejiang 310058 China; ^4^ Liangzhu Laboratory Zhejiang University Medical Center Hangzhou Zhejiang 311121 China; ^5^ Department of Orthopaedic Surgery Sir Run Run Shaw Hospital Zhejiang University School of Medicine Hangzhou Zhejiang 310016 China; ^6^ Department of Orthopaedic Surgery The First Affiliated Hospital of Wenzhou Medical University Wenzhou Zhejiang 325000 China; ^7^ Department of Thyroid and Breast Surgery, Ningbo First Hospital Zhejiang University Ningbo Zhejiang 315010 China; ^8^ School of Medicine Shaoxing University Shaoxing Zhejiang 312000 China

**Keywords:** extracellular matrix stiffness, primary liver cancers, synergistic anticancer strategy

## Abstract

The development of primary liver cancer (hepatocellular carcinoma [HCC] and intrahepatic cholangiocarcinoma [ICC]) is linked to its physical microenvironment, particularly extracellular matrix (ECM) stiffness. Potential anticancer strategies targeting ECM stiffness include prevention/reversal of the stiffening process and disruption of the response of cancer cells to mechanical signals from ECM. However, each strategy has limitations. Therefore, the authors propose integrating them to maximize their strengths. Compared with HCC, ICC has a stiffer ECM and a worse prognosis. Therefore, ICC is selected to investigate mechanisms underlying the influence of ECM stiffness on cancer progression and application of the integrated anticancer strategy targeting ECM stiffness. In summary, immunofluorescence results for 181 primary liver cancer tissue chips (ICC, *n* = 91; HCC, *n* = 90) and analysis of TCGA mRNA‐sequencing demonstrate that ECM stiffness can affect phenotypes of primary liver cancers. The YAP1/ABHD11‐AS1/STAU2/ZYX/p‐YAP1 pathway is a useful entry point for exploration of specific mechanisms of mechanical signal conduction from the ECM in ICC cells and their impact on cancer progression. Moreover, a synergistic anticancer strategy targeting ECM stiffness (ICCM@NPs + siABHD11‐AS1@BAPN) is constructed by integrating ECM softening and blocking intracellular mechanical signal transduction in ICC and can provide insights for the treatment of cancers characterized by stiff ECM.

## Graphical abstract

Extracellular matrix (ECM) stiffness influences phenotypes of primary liver cancers, and the YAP1/ABHD11‐AS1/STAU2/ZYX/p‐YAP1 pathway is a useful entry point to delve into specific mechanisms of mechanical signal transduction from the ECM in intrahepatic cholangiocarcinoma and corresponding effects on cancer progression. Based on these insights, this study devises a synergistic anticancer strategy that integrates ECM softening and mechanical signal transduction blockade.

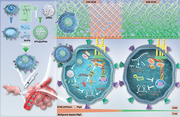

## 1 Background

The extracellular matrix (ECM), which is composed of collagens, fibronectin, glycosaminoglycans, elastin, laminins, and various combinations of these molecules, creates a non‐cellular macromolecular framework in 3D space.^[^ ^1^ ^]^ In the context of solid malignancies, the disruption of proper ECM homeostasis is often considered a defining characteristic.^[^ ^2^, ^3^ ^]^ The ECM is important in cancer biology, providing mechanical support, modulating the microenvironment, and serving as a source of signaling molecules.^[^ ^2^, ^4^ ^]^ There is a growing body of evidence suggesting that the development of cancer is strongly influenced by the physical properties of the ECM, particularly its stiffness.^[^ ^5^, ^6^, ^7^ ^]^ When a tumor develops, the ECM tends to become stiffer. This tendency can be attributed to several factors, including enhanced matrix deposition, collagen cross‐linking, fiber stiffening, elevated cell density, heightened interstitial fluid pressure, and intensified cell‐to‐cell interactions.^[^ ^8^ ^]^ Increased ECM stiffness is not only a common feature of various types of cancer but also an important factor in the progression of cancer because a stiffened ECM amplifies the growth, survival, and migratory capabilities of cancer cells, thereby facilitating epithelial–mesenchymal transition.^[^ ^3^, ^9^ ^]^

Primary liver cancer, which includes hepatocellular carcinoma (HCC) and intrahepatic cholangiocarcinoma (ICC), is a useful model for studying the impact of ECM stiffness in solid malignancies for several reasons. First, the liver is an organ with an abundance of ECM, and the properties of the ECM directly affect the behavior of liver cells.^[^ ^10^ ^]^ Second, liver cancer frequently coincides with the development of hepatic fibrosis, resulting in remodeling and stiffening of the ECM, which alters the microenvironment in which the cancer cells are situated.^[^ ^11^ ^]^ Third, primary liver cancer is one of the most common cancers globally. In 2020, it ranked sixth among newly diagnosed malignancies and was the third leading cause of cancer‐related mortality worldwide.^[^ ^12^, ^13^ ^]^ Remodeling of the ECM can influence mechanical signal transduction in the microenvironment of primary liver cancer. It is widely accepted that chronic liver diseases characterized by abundant deposition of ECM may transmit mechanical pressure to underlying hepatocytes, portal fibroblasts, liver sinusoidal endothelial cells, and hepatic stellate cells. Transient elastography has indicated a significant increase in liver stiffness, escalating from 2.1 kPa to more than 6.0 kPa, with the progression of liver fibrosis or cirrhosis.^[^ ^8^ ^]^ Moreover, in cases of HCC, the stiffness value could reach levels exceeding 20 kPa in livers affected by cancers.^[^ ^14^, ^15^ ^]^ HCC cells cultured on mechanically adjustable “stiff gels” displayed a growth state dependent on mitotic signaling, while cells cultured on “soft gels” exhibited characteristics of stem cells.^[^ ^16^ ^]^ Mechanical sensors on the surface of cancer cells convert mechanical signals in the tumor microenvironment into biological signals, including integrins, linker proteins, membrane‐bound receptor tyrosine kinases, selective G‐protein‐coupled receptors, ion channels, and glycoproteins. Among these receptors, integrins have been extensively studied for their involvement in ECM–cell interactions. Integrins connect ECM with the cell cytoskeleton and activate mechanical signal transduction pathways.^[^ ^17^ ^]^ They also link the cytoskeleton to actomyosin through various adaptor proteins, including talin, vinculin, and paxillin, and mechanically stimulated integrins activate the downstream kinases Src and FAK as well as GTPases, such as Rac and RhoA.^[^ ^18^, ^19^ ^]^ The YAP1 signaling pathway is important in HCC, connecting ECM stiffness and mechanical signaling to the development of cancer.^[^ ^20^ ^]^ In individuals with liver fibrosis, the mechanical pressure caused by a stiffer ECM was found to inhibit the Hippo pathway, resulting in increased levels of YAP1 and its nuclear translocation.^[^ ^21^ ^]^ Within the cell nucleus, YAP1 acts as a transcriptional coactivator, binding to corresponding transcription factors and upregulating a specific set of target genes.

Anticancer strategies based on the molecular mechanisms associated with ECM stiffness are currently under development. Prevention/reversal of ECM stiffening and disruption of the intracellular response of cancer cells to mechanical signals from the ECM might be potential anticancer strategies.^[^ ^22^ ^]^ Given that ECM stiffening depends largely on excessive deposition and cross‐linking within the ECM, it can be prevented or reversed by degrading the ECM and reducing its cross‐linking. β‐aminopropionitrile (BAPN) is a specific lysyl oxidase (LOX) inhibitor, and its mechanism of action involves competitive binding to the active site of the LOX enzyme, preventing it from oxidizing amino acid residues. This action decreases the cross‐linking of collagen fibers, thereby affecting the structure and mechanical properties of the ECM.^[^ ^23^ ^]^ In a previous study, we confirmed that BAPN decreased tissue stiffness and collagen content in HCC.^[^ ^24^ ^]^ Although BAPN can be used to prevent the progression of pathology caused by matrix cross‐linking, it has some limitations that have been impeding its clinical application, including toxicity and the fact that softening or degradation of the ECM eliminates obstacles to the invasion of cancer.^[^ ^25^, ^26^ ^]^ The mechanical constraints of type I collagen can counteract the cancer‐promoting effects of cancer‐associated fibroblasts, which suggests that blindly degrading the ECM may have unintended consequences and that preserving type I collagen while targeting cancer‐associated fibroblasts may be more advantageous.^[^ ^27^ ^]^ As mentioned earlier, the YAP1 signaling pathway is widely considered to play an important role in the intracellular response of cancer cells to mechanical signals from the ECM, and ECM stiffness influences the localization and activity of YAP1 within the cell.^[^ ^20^ ^]^ However, YAP1 is a critical transcription regulatory factor, driving regulation of the shape, migration, and differentiation of cells as well as cell growth and proliferation.^[^ ^28^, ^29^ ^]^ Therefore, directly inhibiting the expression of YAP1 might also affect various aspects of normal cells, and it is necessary to screen for appropriate targets that respond to mechanical signals from the ECM but differentiate between cancer cells and normal cells, so that damage to normal cells can be avoided. It should also be noted that breaking through the stiff ECM is a challenging and problematic aspect of precision molecular therapy within cells. Hence, both the above‐mentioned anticancer strategies have certain limitations when applied alone. To overcome these limitations, we propose integrating these approaches to leverage their individual strengths and foster complementarity.

However, the exploration of mechanisms and the application of treatment strategies in relevant fields have often been based on HCC, and there is limited literature on the relationship between ECM stiffness and the onset and progression of ICC. In‐depth research has shown that the ECM is significantly stiffer in ICC than in HCC,^[^ ^30^, ^31^ ^]^ and that ICC has a higher degree of malignancy and a poorer long‐term prognosis.^[^ ^32^, ^33^, ^34^ ^]^ Therefore, after demonstrating that ECM stiffness could affect phenotypes of primary liver cancers, we used ICC to further explore the specific mechanisms underlying the impact of ECM stiffness on the progression of cancer and the effects of the application of anticancer strategies targeting ECM stiffness. We first screened for functional long non‐coding RNAs (lncRNAs) in response to ECM stiffness (ABHD11‐AS1) and identified it as a previously undiscovered novel transcript in ICC. Next, we used the YAP1/ABHD11‐AS1/STAU2/ZYX/p‐YAP1 signaling pathway as the entry point to explore specific mechanisms via which mechanical signals are conducted from the ECM in ICC cells and their impact on the progression of cancer. We also constructed a synergistic anticancer strategy (ICCM@NPs + siABHD11‐AS1@BAPN) that targeted ECM stiffness by integrating softening of the ECM and blocking of intracellular mechanical signal transduction in ICC with the aim of providing insights for the development of treatment for cancers characterized by a stiff ECM.

## 2 Results

### 2.1 ECM Stiffness Could Affect the Long‐Term Prognosis of Patients with Primary Liver Cancer

**Figure 1:**
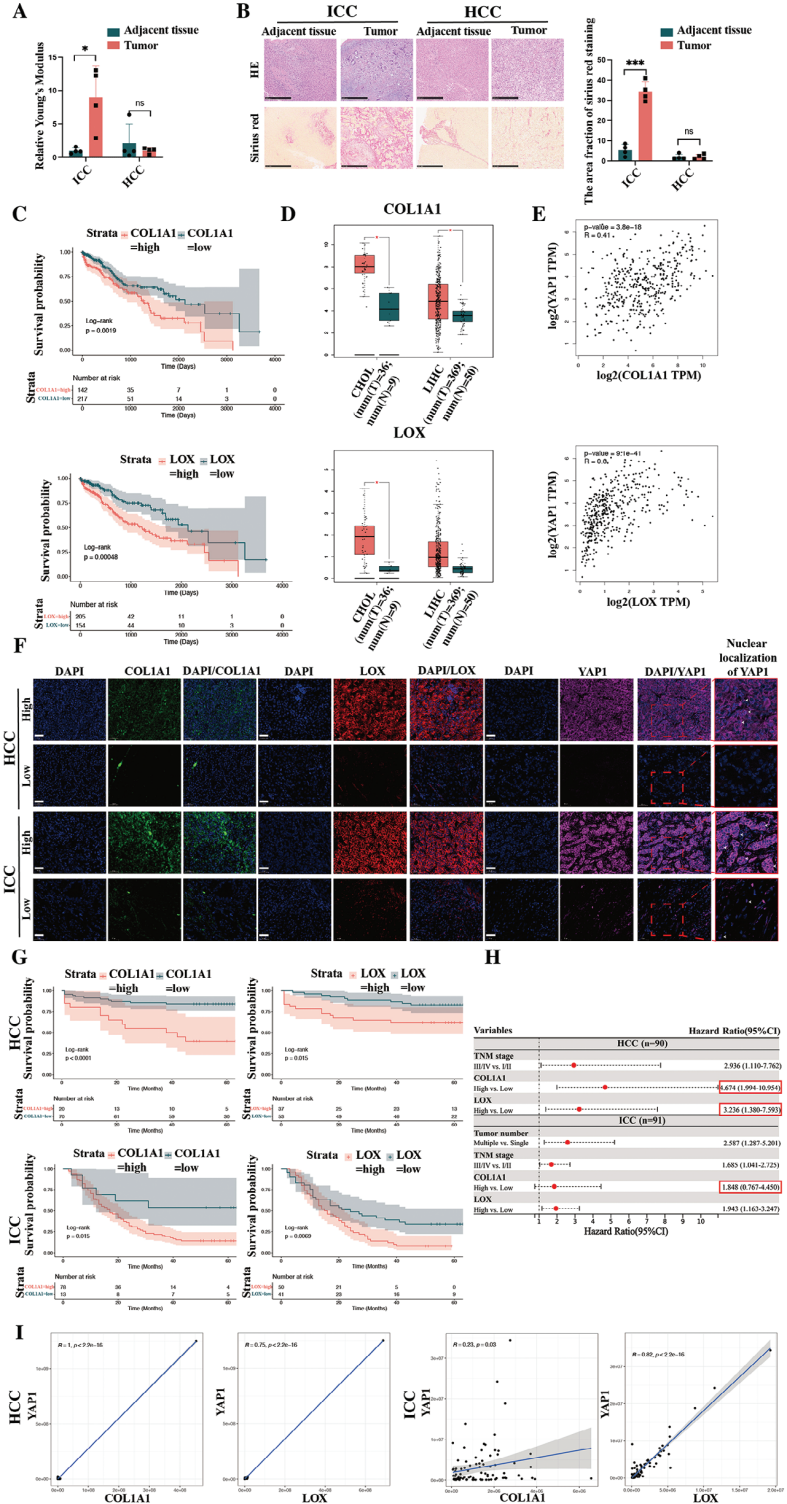
Extracellular matrix (ECM) stiffness could affect the long‐term prognosis of patients suffering primary liver cancer (including HCC and ICC). A) The relative Young's modulus of primary liver cancer (including HCC and ICC) and paired adjacent tissues assessed by atomic force microscope (AFM) (*n* = 4); B) The representative staining images and quantification for collagen content of primary liver cancer (including HCC and ICC) and paired adjacent tissues (sirius red staining (scale bar = 500 µm)) (*n* = 4); C) The impact of collagen‐related molecules (COL1A1, LOX) on long‐term prognosis in patients with primary liver cancer according to TCGA transcriptome sequencing profiling; D) The exploration of the differential expression of collagen‐related molecules (COL1A1, LOX) between primary liver cancer and normal tissues according to TCGA transcriptome sequencing profiling; E) The relationship between collagen‐related molecules (COL1A1, LOX) and ECM stiffness‐related transcription co‐activator YAP1 in primary liver cancer according to TCGA transcriptome sequencing profiling; F) The representative immunofluorescence images of tissue microarray of HCC (*n* = 90, above) and ICC (*n* = 91, below) (immunofluorescence of COL1A1, LOX and YAP1). (scale bar = 50 µm); G) The impact of collagen‐related molecules (COL1A1, LOX) on long‐term prognosis in patients with HCC (above) or ICC (below) according to immunofluorescence of tissue microarray; H) The results of multivariable analyses of overall survival in patients with primary liver cancers; I) The relationship between collagen‐related molecules (COL1A1, LOX) and ECM stiffness‐related transcription co‐activator YAP1 in HCC (left) or ICC (right) according to immunofluorescence of tissue microarray; The continuous variables of normal distribution were represented as the mean ± standard error of the mean. Student's *t*‐test was employed to compare continuous variables following normal distribution (A,B, D). Survival analysis was performed using the Kaplan–Meier method, and survival curves were simultaneously plotted; The log‐rank test was used to compare the different groups' overall survival (C,G). *P*‐value <0.05 was considered statistically significant. *p < 0.05, **p < 0.01, ***p < 0.001.

Basic information regarding the datasets included in the study is shown in **Table** **1**. Atomic force microscopy (AFM) results demonstrated that the Young's modulus of fresh ICC tissue was greater than that of adjacent tissues, while there was no statistically significant difference in the Young's modulus of fresh HCC tissue compared to adjacent tissues (**Figure** **1**A) (*n*[ICC] = *n*[HCC] = four pairs). Sirius red staining revealed that the collagen content of ICC tissue was also higher than that of adjacent tissues. In contrast, there was no statistically significant difference in collagen content between HCC tissue and adjacent tissues (Figure 1B) (*n*[ICC] = *n*[HCC] = four pairs). This study incorporated mRNA sequencing data from The Cancer Genome Atlas (TCGA), including TCGA‐CHOL (*n*[T] = 36, *n*[N] = 9) and TCGA‐LIHC (*n*[T] = 369, *n*[N] = 50) for analysis of the expression levels of collagen‐related molecules and their influence on the long‐term prognosis of primary liver cancers, including HCC and ICC (Figure 1C,E). This analysis revealed that higher levels of COL1A1 and LOX, both of which are collagen‐related molecules, were associated with a worse long‐term prognosis in patients with primary liver cancer. Furthermore, COL1A1 and LOX mRNA levels were higher in ICC tissue than in non‐cancerous tissue. The COL1A1 mRNA level was also higher in HCC tissue than in non‐cancerous tissue but the difference in the LOX mRNA level was not statistically significant. This finding indicated that the difference in expression levels of collagen‐related molecules between ICC and non‐cancerous tissue is more pronounced than that between HCC and non‐cancerous tissue. We also found a significant positive correlation of the mRNA expression levels of collagen‐related molecules in primary liver cancers with the stiffness‐related transcription co‐activator YAP1, suggesting that changes in extracellular collagen content or ECM stiffness could affect the activity of YAP1 and subsequently influence transcriptional activity in primary liver cancer cells.

**Table 1:** Basic information of datasets included in the study.

Datasets	Range of years	Total number of patients	Data type	Usage
SRRSH‐HCC_tissue microarray	from April 2014 to November 2015	90	Tissue microarray	IF
SRRSH‐ICC_tissue microarray	from January 2014 to December 2019	91	Tissue microarray	IF
SRRSH‐ICC_mRNA sequencing	from September 2016 to May 2018	19	mRNA sequencing	Bioinformatics analysis
SRRSH‐HCC_fresh tissue	November 2022	5	Fresh tissue	AFM and IF
SRRSH‐ICC_fresh tissue	November 2022	5	Fresh tissue	AFM and IF
TCGA‐LIHC	From 1995 to 2013	369	mRNA sequencing	Bioinformatics Analysis
TCGA‐CHOL	From 2005 to 2013	36	mRNA sequencing	Bioinformatics Analysis

AFM: atomic force microscope; IF: immunofluorescence.

The corresponding protein‐level changes in collagen‐related molecules were revealed by immunofluorescence of tissue microarrays for 91 ICC cases and 90 HCC cases, and the representative immunofluorescence images of high or low expression of collagen‐related molecules were presented in Figure 1F. Consistent with the results of TCGA data, the immunofluorescence results of the tissue microarrays showed that COL1A1 and LOX influenced the long‐term prognosis of both HCC and ICC and that higher expression levels of these collagen‐related molecules were associated with a worse long‐term prognosis in patients with primary liver cancer (Figure 1G). Cox regression analyses indicated that the expression levels of collagen‐related molecules were independent predictors of long‐term overall survival in these patients (Figure 1H and **Tables** **2**, **3**, **4**). The protein expression levels of collagen‐related molecules in both HCC and ICC showed a significant positive correlation with YAP1, providing protein‐level evidence that changes in extracellular collagen content (ECM stiffness) could affect the activity of YAP1 and subsequently affect transcriptional activity in primary liver cancer cells (Figure 1I).

**Table 2:** Clinical characteristics of 90 HCC patients based on COL1A1 and LOX expression levels.

Variables	All patients	COL1A1		*p*‐value	LOX		*p‐*value
		High expression (20)	Low expression (70)		High expression (37)	Low expression (53)	
Age				0.318			0.408
<60	69	17 (85.0)	52 (74.3)		30 (81.1)	39 (73.6)	
≥60	21	3 (15.0)	18 (25.7)		7 (18.9)	14 (26.4)	
Gender				0.522			0.106
Female	13	2 (10.0)	11 (15.7)		8 (21.6)	5 (9.4)	
Male	77	18 (90.0)	59 (84.3)		29 (78.4)	48 (90.6)	
Tumor number				0.891			0.712
Single	86	19 (95.0)	67 (95.7)		35 (94.6)	51 (96.2)	
Multiple	4	1 (5.0)	3 (4.3)		2 (5.4)	2 (3.8)	
Differentiation				0.027			0.900
Low	4	3 (15.0)	1 (1.4)		2 (5.4)	2 (3.8)	
Moderate/well	84	17 (85.0)	67 (95.7)		34 (91.9)	50 (94.3)	
NA	2	0 (0)	2 (2.9)		1 (2.7)	1 (1.9)	
Lymph node metastasis				0.891			0.712
Yes	4	1 (5.0)	3 (4.3)		2 (5.4)	2 (3.8)	
No	86	19 (95.0)	67 (95.7)		35 (94.6)	51 (96.2)	
Distant metastasis				0.172			0.712
Yes	4	2 (10.0)	2 (2.9)		2 (5.4)	2 (3.8)	
No	86	18 (90.0)	68 (97.1)		35 (94.6)	51 (96.2)	
TNM stage				0.152			0.545
I/II	80	16 (80.0)	64 (91.4)		32 (86.5)	48 (90.6)	
III/IV	10	4 (20.0)	6 (8.5)		5 (13.5)	5 (9.4)	

**Table 3:** Clinical characteristics of 91 ICC patients based on COL1A1 and LOX expression levels.

Variables	All patients	COL1A1		*p*‐value	LOX		*p‐*value
		High expression (78)	Low expression (13)		High expression (50)	Low expression (41)	
Age				0.636			<0.001
<60	26	23 (29.5)	3 (23.1)		7 (14.0)	19 (46.3)	
≥60	65	55 (70.5)	10 (76.9)		43 (86.0)	22 (53.7)	
Gender				0.666			0.401
Female	40	35 (44.9)	5 (38.5)		20 (40.0)	20 (48.8)	
Male	51	43 (55.1)	8 (61.5)		30 (60.0)	21 (51.2)	
Tumor number				0.149			0.500
Single	80	67 (85.9)	13 (100)		45 (90.0)	35 (85.4)	
Multiple	11	11 (14.1)	0 (0)		5 (10.0)	6 (14.6)	
Differentiation				0.306			0.083
Low	55	45 (57.7)	10 (76.9)		26 (52.0)	29 (70.7)	
Moderate/well	31	29 (37.2)	2 (15.4)		22 (44.0)	9 (22.0)	
NA	5	4 (5.1)	1 (7.7)		2 (4.0)	3 (7.3)	
Lymph node metastasis				0.340			0.958
Yes	38	31 (39.7)	7 (53.8)		21 (42.0)	17 (41.5)	
No	53	47 (60.3)	6 (46.2)		29 (58.0)	24 (58.5)	
Distant metastasis				0.489			0.667
Yes	15	12 (15.4)	3 (23.1)		9 (18.0)	6 (14.6)	
No	76	66 (84.6)	10 (76.9)		41 (82.0)	35 (85.4)	
TNM stage				1.000			0.649
I/II	42	36 (46.2)	6 (46.2)		22 (44.0)	20 (48.8)	
III/IV	49	42 (53.8)	7 (53.8)		28 (56.0)	21 (51.2)	

**Table 4:** Univariable and multivariable analyses of overall survival.

Variables	HCC (*n* = 90)						ICC (*n* = 91)					
	Univariable analysis			Multivariable analysis			Univariable analysis			Multivariable analysis		
	HR	95%Cl	*P* value	HR	95%Cl	*P* value	HR	95%Cl	*P* value	HR	95%Cl	*P* value
Age, ≥60 versus <60	0.898	0.333–2.420	0.832				1.578	0.914–2.726	0.102			
Gender, Male versus Female	1.900	0.445–8.104	0.386				1.088	0.684–1.731	0.721			
Tumor number, Multiple versus Single	2.648	0.619–11.322	0.189				2.611	1.355–5.031	0.004	2.587	1.287‐5.201	0.008
Differentiation, Low versus Others	0.878	0.118–6.513	0.898				0.966	0.592–1.576	0.889			
TNM stage, III/IV versus I/II	4.068	1.593–10.389	0.003	2.936	1.110‐7.762	0.030	1.792	1.121–2.867	0.015	1.685	1.041‐2.725	0.034
COL1A1, High versus Low	4.745	2.086–10.791	<0.001	4.674	1.994–10.954	<0.001	2.676	1.155–6.201	0.022	1.848	0.767–4.450	0.171
LOX, High versus Low	2.697	1.166–6.240	0.020	3.236	1.380–7.593	0.007	1.912	1.177–3.106	0.009	1.943	1.163–3.247	0.011

### 2.2 ECM Stiffness Could Affect the Malignant Phenotype, Intracellular Mechanical Conduction, and Nuclear Transcriptional Activity in ICC and HCC Cells

**Figure 2:**
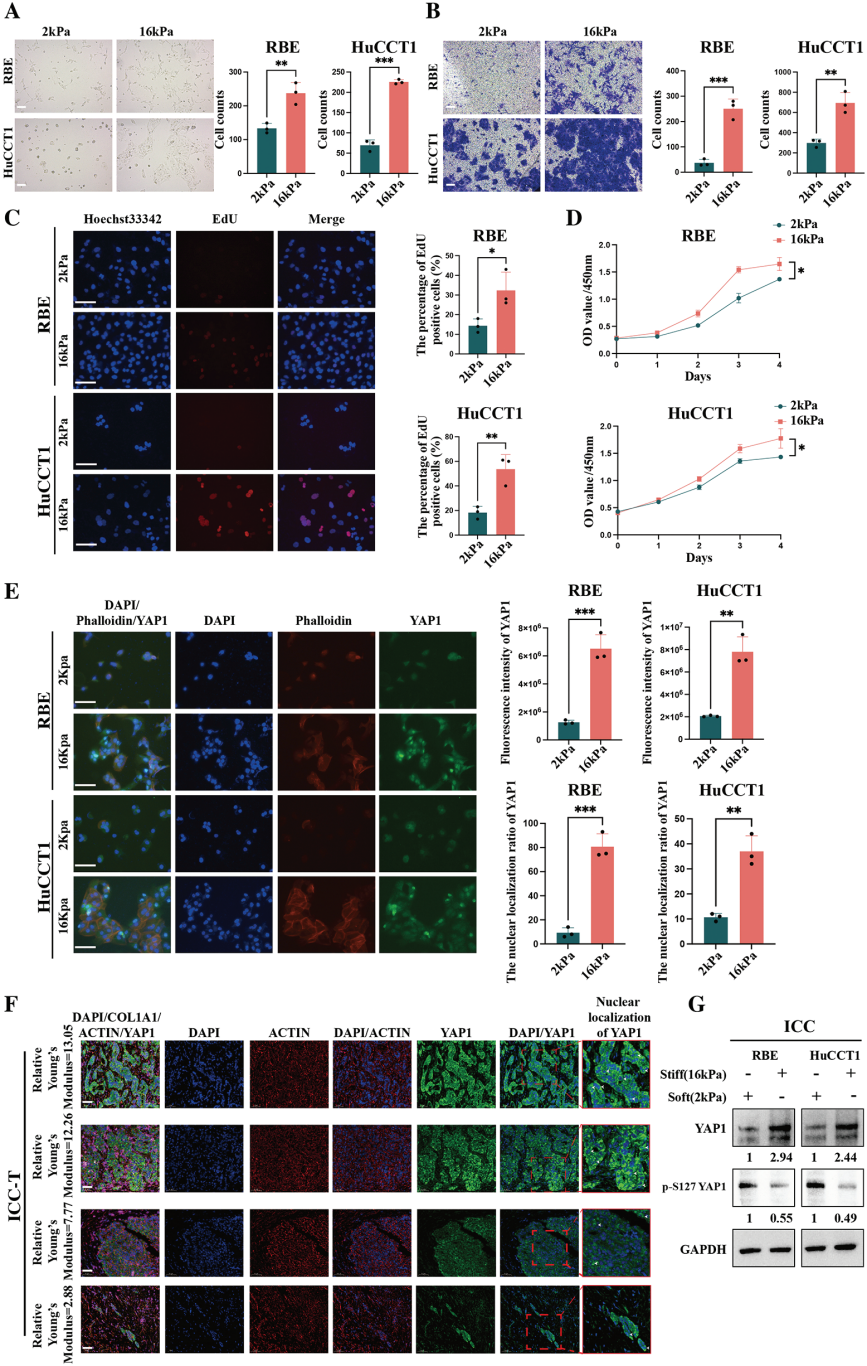
ECM stiffness could affect the malignant phenotype, intracellular mechanical conduction, and nuclear transcriptional activity of ICC cells; A–D) Cell experiments exhibited that ECM stiffness (16 kPa vs 2 kPa) could affect the malignant phenotype of ICC cells–cell morphology under optical microscopy (scale bar = 50 µm), Invasion (scale bar = 50 µm), EdU assays (scale bar = 100 µm) (The time points for the assay were chosen at 48 h after treatment) and CCK‐8 assays (*n* = 3); E) The multicolor fluorescence images of DAPI/Phalloidin/YAP1 in ICC cells under different ECM stiffness conditions (16 kPa vs 2 kPa) (scale bar = 100 µm) (The time points for the assay were chosen at 48 h after treatment) (*n* = 3); F) The multicolor immunofluorescence images of DAPI/COL1A1/ACTIN/YAP1 in ICC tissues under different ECM stiffness conditions (scale bar = 50 µm); G) The western blot assays showing that ECM stiffness (16 kPa vs 2 kPa) could affect the content and activity of YAP in ICC (The time points for the assay were chosen at 48 h after treatment). The continuous variables of normal distribution were represented as the mean ± standard error of the mean. Student's *t*‐test was employed to compare continuous variables following normal distribution (A–D). *P*‐value < 0.05 was considered statistically significant. *p < 0.05, **p < 0.01, ***p < 0.001.

In the cell experiments, 6‐well CytoSoft plates with differences in Young's modulus (2 and 16 kPa) were used to simulate a difference in ECM stiffness. **Figures** **2**A and S1A (Supporting Information; *n* = 3) showed that the number of ICC cells (RBE and HuCCT1) or HCC cells (SK and G2) was significantly greater on the 16 kPa plate than on the 2 kPa plate and that the cell status was better on the 16 kPa plate. Invasion assays, EdU assays, and CCK8 assays demonstrated that the ability of ICC cells (RBE and HuCCT1) to invade and proliferate was greater on the 16 kPa plate than on the 2 kPa plate (Figure 2B–D; *n* = 3), which suggested that ECM stiffness could affect the malignant phenotypes of primary liver cancer cells at the cell level. Furthermore, both immunofluorescence staining of tissues and cell experiments showed that ECM stiffness could influence the intracellular mechanical conduction and nuclear transcriptional activity of primary liver cancers. The stiffness of the ECM was positively correlated with the regularity of actin arrangement, the content of YAP1, and the nuclear localization ratio of YAP1 (Figure 2E,F, Figure S1B,C, Supporting Information). Western blot assays demonstrated that the total YAP content in primary liver cancer cells (including both HCC and ICC) was significantly higher on the 16 kPa plate, while the p‐S127 YAP1 content was significantly higher on the 2 kPa plate (Figure 2G; Figure S1D, Supporting Information). Considering that the ECM is significantly stiffer in ICC than in HCC, that ICC shows a higher degree of malignancy and has a poorer long‐term prognosis, and the lack of information in the literature on the relationship between ECM stiffness and the onset and progression of ICC, we used ICC as an example to explore the relationship between ECM stiffness and the onset and development of primary liver cancer.

### 2.3 Screening of Functional lncRNAs Related to ECM Stiffness in ICC (Anchored as ABHD11‐AS1), Its Phenotypes, and Corresponding Mechanisms of ABHD11‐AS1 Regulated by ECM Stiffness

**Figure 3:**
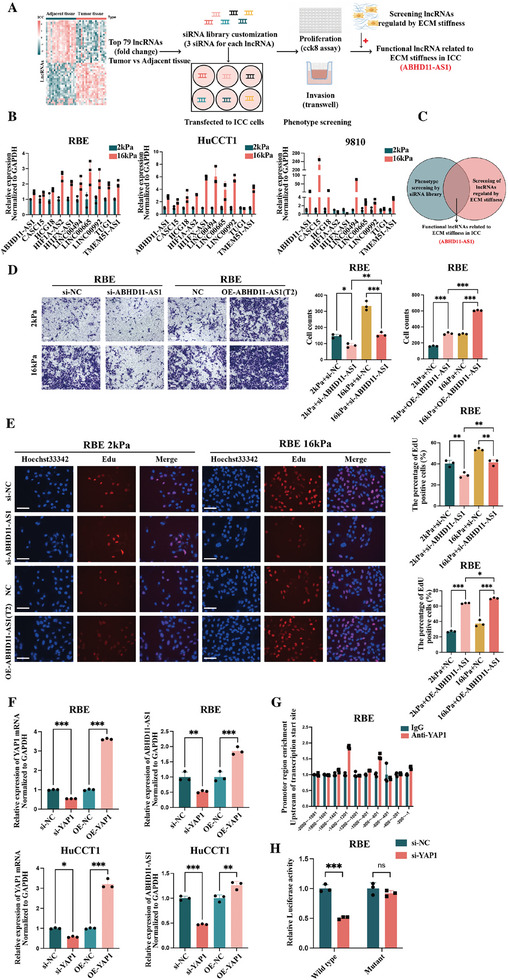
Screening of functional lncRNAs related to ECM stiffness in ICC (anchored as ABHD11‐AS1), phenotypes and corresponding mechanisms of ABHD11‐AS1 regulated by ECM stiffness; A) The schematic diagram of screening functional lncRNAs related to ECM stiffness; B) The screening of lncRNAs regulated by ECM stiffness, the changes in lncRNAs expression levels caused by culture dishes covered with functionalized silicone of different stiffness (The time points for the assay were chosen at 48 h after treatment) (*n* = 3); C) The results of the screening of functional lncRNAs related to ECM stiffness—finally anchoring ABHD11‐AS1; D,E) The phenotypes of the knockdown or overexpression of ABHD11‐AS1 in RBE cells under culture dishes covered with functionalized silicone of different stiffness, including invasion (transwell, scale bar = 50 µm) and proliferation (EdU staining, scale bar = 100 µm) (The time points for the assay were chosen at 48 h after treatment) (*n *= 3); F) The expression of ABHD11‐AS1 when YAP1 was knocked down or overexpressed in ICC cell lines (The time points for the assay were chosen at 48 h after treatment) (*n* = 3); G) The results of chip assay revealing the promotor region of ABHD11‐AS1(the transcript T2), which YAP1 could bind to (*n* = 3); H) The results of dual renilla and firefly luciferase reporter assays using the linear form of wild‐type and mutant‐type ABHD11‐AS1 promotor in RBE cells (*n *= 3). The continuous variables of normal distribution were represented as the mean ± standard error of the mean. Student's *t‐*test was employed to compare continuous variables following normal distribution (B,F–H). The analysis of variance (ANOVA) test was employed for comparing data of multiple groups (D,E). *P‐*value <0.05 was considered statistically significant. *p < 0.05, **p < 0.01, ***p < 0.001.

A flow chart showing the procedure used to screen for functional long non‐coding RNAs (lncRNAs) related to ECM stiffness in ICC is shown in **Figure** **3**A. The screening procedure was as follows. First, transcriptome sequencing was performed for 19 pairs of ICC and corresponding adjacent tissues. After transcriptome sequencing and data preprocessing, the lncRNAs were identified and annotated with known sequences using lncRNA databases, including GENCODE, NONCODE, and LncRNADisease.^[^ ^35^, ^36^, ^37^ ^]^ Next, the top 79 lncRNAs (according to the fold change of tumors vs adjacent tissues) were selected for subsequent experiments, including cell phenotype screening and screening of lncRNAs related to ECM stiffness in ICC. The cell phenotype screening experiment was performed with small interfering RNA (siRNA) libraries (three siRNAs per lncRNA) and included proliferation and invasion assays. Figure S2A (Supporting Information; *n* = 3) showed the results of the CCK8 assay for the top 15 siRNAs (corresponding to 10 lncRNAs) that influenced the proliferation ability of ICC cells. Figure S2B (Supporting Information) showed the impact of the above‐mentioned top 15 siRNAs on the invasion ability of ICC cells (the five siRNAs with the most significant differences in invasion assays were si‐ABHD11‐AS1_001, si‐HIF1A‐AS2_002, si‐H1FX‐AS1_002, si‐ABHD11‐AS1_002, and si‐ABHD11‐AS1_003). The screening experiment for lncRNAs related to ECM stiffness in ICC explored the differences in RNA levels of the aforementioned 10 lncRNAs of ICC cells cultivated on plates with different Young's modulus (2 and 16 kPa). Seven lncRNAs (ABHD11‐AS1, CASC15, HCG18, LINC00494, LINC00665, LINC00992, and TMEM51‐AS1) were found to be affected by ECM stiffness, and their RNA levels increased in ICC cells on the 16 kPa plates (Figure 3B; *n* = 3). The intersection of results obtained from screening of cell phenotypes and lncRNAs related to ECM stiffness in ICC corresponded to functional lncRNAs related to ECM stiffness in ICC. Ultimately, ABHD11‐AS1 was identified as our screening target (Figure 3C). A volcano plot was created to show the differential lncRNAs between ICC and adjacent tissues and the location of ABHD11‐AS1 according to the transcriptome sequencing for 19 patients (Figure S3A, Supporting Information). Survival analysis of TCGA‐CHOL demonstrated that the long‐term prognosis of ICC was worse in patients with high ABHD11‐AS1 expression than in those with low ABHD11‐AS1 expression (Figure S3B, Supporting Information).

The results of the RACE (Rapid‐Amplification of cDNA Ends) experiment indicated that the transcript sequence of ABHD11‐AS1 in two ICC cell lines (RBE and HuCCT1) differed from the sequence found on the NCBI official website, so could be defined as a novel transcript sequence. Specifically, the transcript ABHD11‐AS1‐T1 was only expressed in the RBE cell line, while the transcript ABHD11‐AS1‐T2 was expressed in the RBE and HuCCT1 cell lines. Therefore, the common transcript ABHD11‐AS1‐T2 in the RBE and HuCCT1 cell lines was used for subsequent experiments (Figure S3C, Table S4, Supporting Information). Quantitative reverse transcription polymerase chain reaction (qRT‐PCR) assays revealed that transfection of plasmids containing the corresponding transcripts showed favorable efficiency in terms of overexpressing ABHD11‐AS1 (Figure S3D, Supporting Information; *n *= 3).

Figure S3E (Supporting Information; *n* = 3) demonstrated that the expression levels of ABHD11‐AS1 were upregulated in three ICC cell lines (RBE, HuCCT1, and 9810) in comparison with those in a normal biliary epithelial cell line (HIBEpiC). Phenotype experiments demonstrated that targeting ABHD11‐AS1 with three different siRNAs could inhibit proliferation and invasion to varying degrees in the three ICC cell lines (RBE, HuCCT1, and 9810), with si‐ABHD11‐AS1_003 showing the most stable and significant inhibitory effect (Figure S3F,G, Supporting Information; *n *= 3). In the RBE cell line, overexpression of the ABHD11‐AS1‐T1 or ABHD11‐AS1‐T2 transcript promoted proliferation and invasion. However, in the HuCCT1 cell line, only overexpression of the ABHD11‐AS1‐T2 transcript was associated with increased proliferation and invasion (Figure S3H,I, Supporting Information; *n *= 3).

Invasion and EdU assays demonstrated that overexpression of ABHD11‐AS1‐T2 could partially rescue the inhibitory effect of the soft (2 kPa) plate on RBE invasion and proliferation phenotypes, while si‐ABHD11‐AS1 could partially rescue the promoting effect of the stiff (16 kPa) plate on RBE invasion and proliferation phenotypes (Figure 3D,E; *n* = 3). Considering the more significant impact of regulating ABHD11‐AS1 expression on the phenotype of ICC cell lines cultured on the stiffer plate (Figure 3D,E; *n* = 3), the function and mechanisms of ABHD11‐AS1 were then investigated on the stiffer plate. Given that YAP1 is the crucial stiffness‐related transcription co‐activator, we hypothesized that it could respond to changes in ECM stiffness and regulate transcription of a novel ABHD11‐AS1 transcript (i.e., ABHD11‐AS1‐T2). We knocked down and overexpressed YAP1 in RBE and HuCCT1 cells, and qRT‐PCR assays indicated that expression of ABHD11‐AS1 showed a corresponding decrease and increase, confirming that YAP1 positively regulated expression of ABHD11‐AS1 (Figure 3F; *n* = 3). Considering that YAP1 often regulates the transcription of genes by nuclear translocation, we used Chip assays to explore the YAP1 binding sequences on the promoter region of ABHD11‐AS1‐T2 (i.e., −1400–−1201 and −800–−601) (Figure 3G; *n* = 3). Luciferase assays further confirmed that after deleting the binding sequence of the above‐mentioned promoter region (defined as the mutant sequence), si‐YAP1 no longer reduced the luciferase fluorescence value in ICC cell lines in comparison with si‐NC (the siRNA control) (Figure 3H; *n *= 3).

### 2.4 ZYX Is a Novel Target of ABHD11‐AS1

**Figure 4:**
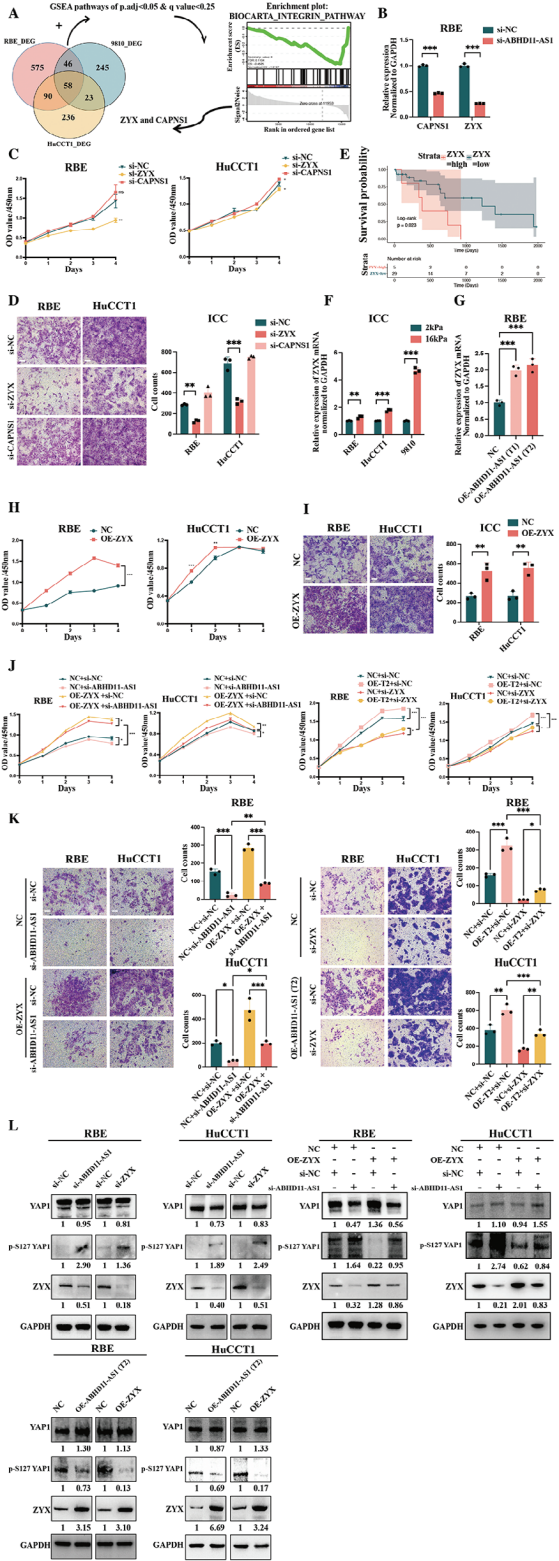
ZYX might be a novel target of ABHD11‐AS1; A) The screening downstream molecules of ABHD11‐AS1 through transcriptome sequencing profiling (preliminary screening identified ZYX and CAPNS1); B) The changes in mRNA of ZYX and CAPNS1 after ABHD11‐AS1 knockdown (validation of transcriptome sequencing results) (The time points for the assay were chosen at 48 h after treatment) (*n *= 3); C,D) The impact of transfecting si‐ZYX or si‐CAPNS1 on proliferation (The time points for the assay were chosen at 0, 1, 2, 3, and 4 days after treatment) and invasion (transwell, scale bar = 50 µm) (The time points for the assay were chosen at 48 h after treatment) abilities of ICC cell lines (ultimately anchored as ZYX) (*n* = 3); E) The impact of ZYX mRNA levels on long‐term prognosis of ICC (based on TCGA‐CHOL data); F) The effect of changing ECM stiffness on ZYX mRNA levels in ICC cells (considering ZYX as a crucial molecule in the BIOCARTA_INTEGRIN_PATHWAY) (The time points for the assay were chosen at 48 hours after treatment) (*n* = 3); G) The impact of overexpressing different transcripts of ABHD11‐AS1 on ZYX mRNA levels of RBE cells (The time points for the assay were chosen at 48 h after treatment) (*n* = 3); H,I) The effect of overexpressing ZYX mRNA on proliferation (The time points for the assay were chosen at 0, 1, 2, 3, and 4 days after treatment) and invasion (transwell, scale bar = 50 µm) (The time points for the assay were chosen at 48 h after treatment) abilities of ICC cell lines (*n* = 3); J,K) The partial rescue of the effects of ABHD11‐AS1 overexpression or knockdown on proliferation (The time points for the assay were chosen at 0, 1, 2, 3, and 4 days after treatment) and invasion (transwell, scale bar = 50 µm) (The time points for the assay were chosen at 48 h after treatment) abilities of ICC cell lines through ZYX knockdown or overexpression (overexpression of T2 transcript) (*n* = 3); L) The partial rescue of the effects of ABHD11‐AS1 knockdown on related protein levels (ZYX and p‐YAP1 proteins) in ICC cell lines through ZYX overexpression (The time points for the assay were chosen at 48 hours after treatment) (*n* = 3). The continuous variables of normal distribution were represented as the mean ± standard error of the mean. Student's *t*‐test was employed to compare continuous variables following normal distribution (B,F,H,I). The ANOVA test was employed for comparing data of multiple groups (C,D,G,J,K). Survival analysis was performed using the Kaplan–Meier method, and survival curves were simultaneously plotted. The log‐rank test was used to compare the different groups' overall survival (E). *P*‐value < 0.05 was considered statistically significant. *p < 0.05, **p < 0.01, ***p < 0.001.

The steps for screening the downstream target mRNA of ABHD11‐AS1 were as follows. First, mRNA sequencing technology was used to detect differential genes (RBE_DEG, HuCCT1_DEG, and 9810_DEG) between si‐ABHD11‐AS1_003 and si‐NC in three ICC cell lines (RBE, HuCCT1, and 9810). The intersection of RBE_DEG, HuCCT1_DEG, and 9810_DEG yielded a total of 58 genes. Next, gene set enrichment analysis was performed based on the mRNA sequencing results for si‐ABHD11‐AS1_003 versus si‐NC, and pathways with an adjusted *p*‐value of <0.05 and a *q*‐value of <0.25 were selected with the corresponding gene sets. Finally, the intersection of the genes selected in the previous two steps resulted in the BIOCARTA_INTEGRIN_PATHWAY and two genes (ZYX and CAPNS1) (**Figure** **4**A). It is worth noting that the BIOCARTA_INTEGRIN_PATHWAY is related to the response of ICC cells to mechanical signals from the ECM. Knocking down ABHD11‐AS1 led to a corresponding decrease in ZYX and CAPNS1 mRNA levels, consistent with the mRNA sequencing results (Figure 4B; *n* = 3). After transfection of si‐ZYX and si‐CAPNS1 in the RBE and HuCCT1 cell lines, compared with si‐NC, si‐ZYX significantly inhibited proliferation and invasion of RBE and HuCCT1 cells, while si‐CAPNS1 showed no inhibitory function (Figure 4C,D; *n* = 3). Considering that the phenotypic direction when ZYX mRNA was knocked down was consistent with that when ABHD11‐AS1 was knocked down, the interaction between ABHD11‐AS1 and ZYX was investigated further. Survival analysis of TCGA‐CHOL demonstrated that the long‐term prognosis of ICC was worse in patients with high ZYX mRNA expression than in those with low ZYX mRNA expression (Figure 4E). Expression of ZYX mRNA was found to be influenced by ECM stiffness, with an increase in the ZYX mRNA level observed in ICC cells cultured on the 16 kPa plate (Figure 4F; *n* = 3). When ABHD11‐AS1 was overexpressed, there was a corresponding increase in ZYX mRNA, indicating that ZYX mRNA was downstream of ABHD11‐AS1 regulation (Figure 4G; *n* = 3).

Furthermore, unlike the control group, overexpressing ZYX promoted proliferation and invasion phenotypes in the RBE and HuCCT1 cell lines (Figure 4H,I; *n* = 3). Rescue experiments for the cancer phenotypes demonstrated that OE‐ZYX could partially rescue the inhibitory effect of si‐ABHD11‐AS1 on the proliferation and invasion ability of RBE and HuCCT1 cells and that si‐ZYX could partially rescue the promoting effect of OE‐ABHD11‐AS1‐T2 on proliferation and invasion (Figure 4J,K; *n* = 3). Previous studies have shown that knocking down ZYX mRNA promoted phosphorylation of YAP1, inhibiting its entry into the nucleus and its functional role.^[^ ^38^ ^]^ Considering that YAP1 is the crucial stiffness‐related transcription co‐activator, the western blot assays focused mainly on the changes in YAP1 and p‐YAP1. In the actual cell experiments, the change in p‐YAP1 was significant but the change in total YAP1 was not immediately obvious. p‐YAP1 levels were increased by si‐ZYX and si‐ABHD11‐AS1 but decreased by overexpression of ZYX and ABHD11‐AS1. Rescue experiments using western blot assays demonstrated that OE‐ZYX exhibited a trend of partially rescuing the increase in p‐YAP1 caused by si‐ABHD11‐AS1 in RBE and HuCCT1 cells (Figure 4L; Figure S6, Supporting Information) (*n *= 3).

### 2.5 Effects of ABHD11‐AS1 on Proliferation and Metastasis In Vivo

**Figure 5:**
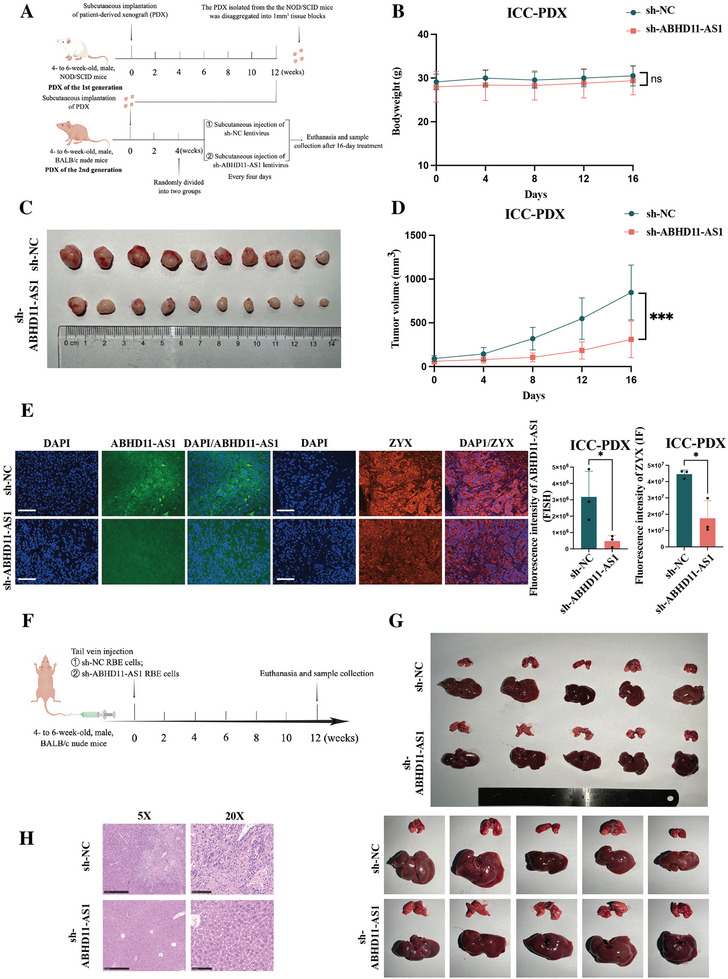
Effects of ABHD11‐AS1 on the proliferation and metastasis in vivo. A) The schematic diagram of the animal experiment reflecting the phenotype of proliferation; B) The comparison of bodyweight change of two groups during the whole course of experimental timeline (*n* = 10); C) The comparison of cancer size of two groups at the end of the experiment (At the 16 days after treatment) (*n *= 10); D) The growth curve of PDX in vivo measured every four days (The time points for the assay were chosen at 0, 4, 8, 12, and 16 days after treatment) (*n* = 10); E) The expression of ABHD11‐AS1 detected by FISH (scale bar = 100 µm), as along with ZYX detected by immunofluorescence (scale bar = 100 µm) in two groups (*n* = 3); F) The schematic diagram of the animal experiment reflecting the phenotype of metastasis; G) The images showing two groups of liver metastases. The metastatic lesions were indicated by the arrows (At the 12 weeks after treatment) (*n* = 5); H) The representative HE staining images of liver metastatic lesions. The continuous variables of normal distribution were represented as the mean ± standard error of the mean. Student's *t*‐test was employed to compare continuous variables following normal distribution (B,D,E). *P*‐value < 0.05 was considered statistically significant. *p < 0.05, **p < 0.01, ***p < 0.001.

We used a patient‐derived tumor xenograft (PDX) mouse model that closely resembles the clinical situation for our in vivo investigation of the role of ABHD11‐AS1 in the progression of ICC. Peritumoral injections of lentivirus expressing sh‐ABHD11‐AS1 or negative control lentivirus were administered every 4 days. After 16 days of treatment, the mice were euthanized (**Figure** **5**A). During the course of the experimental timeline, there was no significant difference in bodyweight change between the two groups (Figure 5B; *n* = 10). While the tumor volume in the sh‐ABHD11‐AS1‐treated group was found to be significantly smaller than that in the control group (Figure 5C,D; *n* = 10). A fluorescence in situ hybridization (FISH) assay of tumor tissues revealed a significant reduction in expression of ABHD11‐AS1 following treatment with sh‐ABHD11‐AS1 lentivirus. Furthermore, the immunofluorescence assay of tumor tissues demonstrated a significant decrease in expression of ZYX after treatment with sh‐ABHD11‐AS1 lentivirus (Figure 5E; *n* = 3). These findings suggested that sh‐ABHD11‐AS1 also inhibited tumor proliferation in vivo.

Next, we injected stably transfected sh‐ABHD11‐AS1 RBE cells and control cells into the tail vein (Figure 5F). After 3 months, significant metastatic lesions in the liver were observed in the control group, while no noticeable intrahepatic metastatic lesions were found in the sh‐ABHD11‐AS1 group (Figure 5G,H; *n *= 5). These findings strongly suggested that the knockdown of ABHD11‐AS1 in ICC cells led to the inhibition of their invasiveness and metastatic potential in vivo.

### 2.6 Mechanisms of the Positive Regulatory Effect of ABHD11‐AS1 on ZYX mRNA

**Figure 6:**
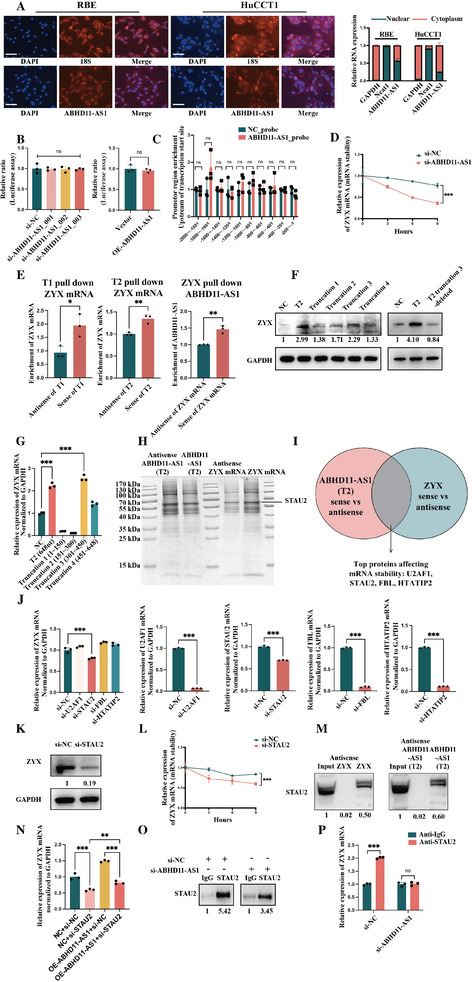
Mechanisms of positive regulation of ABHD11‐AS1 on ZYX mRNA; A) The localization of ABHD11‐AS1 in ICC cells (scale bar = 100 µm) and the results of nuclear‐cytoplasmic separation assays of ICC cells (*n* = 3); B) The results of dual renilla and firefly luciferase reporter assays using the linear form of the promoter region of ZYX in RBE cells after knockdown or overexpression of ABHD11‐AS1 (transcript T2) (*n* = 3); C) The results of Chirp assay showing the binding of ABHD11‐AS1_probe with the promoter region of ZYX mRNA (*n* = 3); D) The results of mRNA stability assays of ZYX mRNA after knockdown of ABHD11‐AS1 using actinomycin D (The time points for the assay were chosen at 0, 3, 6, and 9 h after treatment) (*n *= 3); E) The results of pull‐down assays using RNAmax‐T7 Biotin Labeling Transcription Kit to transcribe the sense and antisense strands of ABHD11‐AS1_T1, ABHD11‐AS1_T2, and ZYX mRNA in vitro (*n* = 3); F,G) The exploration of the specific binding sequence or functional sequence between ABDH11‐AS1 (transcript T2) and ZYX mRNA (The time points for the assay were chosen at 48 h after treatment) (*n* = 3); H) The protein mass spectrometry assays on the pull‐down products obtained using the sense and antisense strands of ABHD11‐AS1_T2 and ZYX mRNA; I) The schematic diagram of screening for (RNA‐binding protein) RBP that could simultaneously bind to ABHD11‐AS1_T2 and ZYX mRNA and affect mRNA stability (resulting in U2AF1, STAU2, FBL, and HTATIP2); J,K) The changes in ZYX mRNA and protein levels after knockdown of RBPs (ultimately anchored as STAU2) (The time points for the assay were chosen at 48 h after treatment) (*n* = 3); L) The results of mRNA stability assays of ZYX mRNA after knockdown of STAU2 mRNA using actinomycin D (The time points for the assay were chosen at 0, 3, 6, and 9 h after treatment) (*n* = 3); M) The results of western blot assays on the pull‐down products obtained using the sense and antisense strands of ABHD11‐AS1_T2 and ZYX mRNA, targeting STAU2 protein for validation of mass spectrometry results; N) The partial rescue of the elevation of ZYX mRNA caused by OE‐ABHD11‐AS1 through the knockdown of STAU2 (The time points for the assay were chosen at 48 h after treatment) (*n* = 3); O) The validation of RIP assays using western blot; P) The results of RIP assays showing that knockdown of ABHD11‐AS1 significantly inhibited the ability of STAU2 to bind to ZYX mRNA (*n* = 3). *p < 0.05, **p < 0.01, ***p < 0.001. The continuous variables of normal distribution were represented as the mean ± standard error of the mean. Student's *t*‐test was employed to compare continuous variables following normal distribution (B–E,J,L,P). The ANOVA test was employed for comparing data of multiple groups (F,N). *P‐*value < 0.05 was considered statistically significant. *p < 0.05, **p < 0.01, ***p < 0.001.

The mRNA levels of ZYX were primarily determined by the rate of synthesis of mRNA (corresponding to nuclear mechanisms) and the mRNA degradation rate (corresponding to cytoplasmic mechanisms). An increase in synthesis or a decrease in degradation could lead to the accumulation of mRNA, resulting in elevated mRNA levels. As previously mentioned, ABHD11‐AS1 positively regulated the expression of ZYX mRNA. The FISH and nuclear–cytoplasmic fractionation experiments simultaneously demonstrated that ABHD11‐AS1 was expressed in both the nucleus and cytoplasm of RBE and HuCCT1 cells (**Figure** **6**A; *n* = 3). Therefore, we hypothesized that ABHD11‐AS1 promoted either transcription of ZYX in the nucleus or stability of ZYX mRNA in the cytoplasm. Exploration of the nuclear mechanisms was divided into two stages. First, dual‐luciferase reporter assays were conducted. The promoter region of ZYX was integrated into the pGL3‐basic plasmid that contained the firefly luciferase. This plasmid was co‐transfected with the pRL‐TK plasmid, which contained the Renilla luciferase gene, into RBE cells. Knockdown and overexpression of ABHD11‐AS1 were then performed in these cells. The findings from the dual‐luciferase reporter assays revealed that neither si‐ABHD11‐AS1 nor OE‐ABHD11‐AS1 significantly modulated the activity of firefly luciferase when compared to the respective control groups, essentially indicating no significant impact on the activity of the ZYX promoter (Figure 6B; *n *= 3). Second, the results of chromatin isolation by RNA purification revealed that the ABHD11‐AS1_probe did not pull down any ZYX promoter region to a statistically significant extent when compared with the NC_probe (Figure 6C; *n* = 3). Therefore, it could be concluded that ABHD11‐AS1 did not promote transcription of ZYX in the nucleus to increase ZYX mRNA levels.

The results of our exploration of the cytoplasmic mechanism were as follows. Actinomycin D assays revealed that compared with transfection using si‐NC, transfection with si‐ABHD11‐AS1 resulted in a gradual decrease in the mRNA levels of ZYX over time, which was consistent with the hypothesized cytoplasmic mechanism (Figure 6D; *n* = 3). Pull‐down experiments were conducted using an RNAmax‐T7 Biotin Labeling Transcription Kit to transcribe the sense and antisense strands of ABHD11‐AS1‐T1, ABHD11‐AS1‐T2, and ZYX mRNA in vitro. When the RBE cell line was used as the RNA source, the sense strand of ABHD11‐AS1‐T1 and ABHD11‐AS1‐T2 transcripts could pull down more ZYX mRNA with statistically significant differences compared to the corresponding antisense strands. Similarly, the sense strand of ZYX mRNA could also pull down more ABHD11‐AS1 with statistically significant differences compared to the corresponding antisense strand (Figure 6E; *n* = 3). These experiments demonstrated interactions between ABHD11‐AS1 and ZYX mRNA in the cytoplasm. After transfection of truncated plasmids and a mutant (T2 truncation 3‐deleted) plasmid, qRT‐PCR, and western blot assays indicated that T2 truncation 3 (301–450) might be the critical sequence needed for ABHD11‐AS1 to stabilize ZYX mRNA (Figure 6F,G; *n* = 3).

LncRNAs have been found to interact with RNA‐binding proteins (RBPs) and to affect both the degradation and stability of mRNAs.^[^ ^39^, ^40^ ^]^ In this study, we performed protein mass spectrometry experiments on the products obtained after the pull‐down of the sense and antisense strands of ABHD11‐AS1‐T2 and ZYX mRNA (Figure 6H, Table S5, Supporting Information). By comparing the intersecting RBPs between ABHD11‐AS1‐T2 sense versus antisense and ZYX mRNA sense versus antisense, we identified the top RBPs that affect mRNA stability to be U2AF1, STAU2, FBL, and HTATIP2 (Figure 6I). When the mRNAs of RBPs were successfully knocked down, we observed a significant decrease in the mRNA level of ZYX only in the si‐STAU2 group in comparison with the si‐NC group (Figure 6J; *n* = 3). Western blot assays also showed that only the si‐STAU2 group showed a significant decrease in the ZYX protein level in comparison with the si‐NC group (Figure 6K). Furthermore, actinomycin D assays demonstrated that transfection with si‐STAU2 resulted in a gradual decrease in the mRNA levels of ZYX over time when compared with transfection using si‐NC (Figure 6L; *n* = 3), which was consistent with the hypothesized cytoplasmic mechanism. Therefore, we focused on STAU2 as the RBP involved in stabilizing ZYX mRNA through its interaction with ABHD11‐AS1. Western blot assays of the products obtained after pull‐down of the sense and antisense strands of ABHD11‐AS1‐T2 and ZYX mRNA confirmed the simultaneous binding of STAU2 to ABHD11‐AS1‐T2 and ZYX mRNA (Figure 6M), thereby confirming the accuracy of the mass spectrometry results. Si‐STAU2 had the potential to partially rescue elevation of ZYX‐mRNA caused by OE‐ABHD11‐AS1 (Figure 6N; *n* = 3). The RNA immunoprecipitation assays demonstrated that transfection of si‐ABHD11‐AS1 significantly inhibited the ability of STAU2 to bind to ZYX mRNA (Figure 6O,P; *n* = 3). In summary, STAU2 functioned as an RBP in the cytoplasm and cooperated with ABHD11‐AS1 to stabilize ZYX mRNA, thereby inhibiting its degradation.

### 2.7 Construction of a Selective Delivery System That Blocked Intracellular Transduction of Mechanical Signals

**Figure 7:**
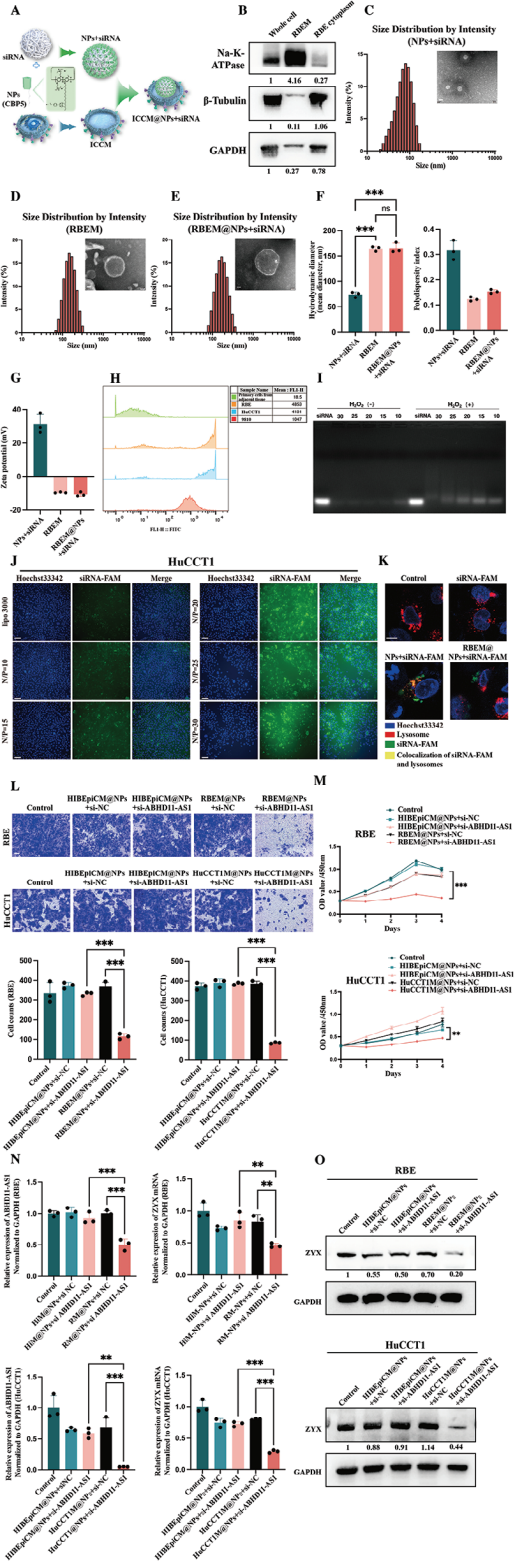
The construction of selective delivery system blocking intracellular mechanical signal transduction. A) The schematic diagram of siRNA loading into CBP5 (NPs) with camouflage by ICCM (ICC cell membrane) coating; B) The results of western blot for membrane protein Na‐K‐ATPase and plasma protein β‐Tubulin, GAPDH in isolated RBEM (RBE cell membrane), RBE cytoplasm, and Whole cell (RBE cell lysate) (proteins of the same quality); C–E) Size and transmission electron microscopy micrograph of NPs+siRNA, RBEM and RBEM@NPs+siRNA (scale bar = 50 nm); F) The particle diameter with the highest frequency of NPs+siRNA, RBEM and RBEM@NPs+siRNA (*n *= 3); G) The zeta potential of NPs+siRNA, RBEM, and RBEM@NPs+siRNA (*n* = 3); H) The results of flow cytometry detecting ROS in different cells; I) The results of gel retardation assay of NPs+siRNA at designated N/P ratios after 1 hour of incubation with or without H_2_O_2_ at 37 °C; J) The visualization of the transfection efficiency in HuCCT1 cells after coculture with lipo3000+siRNA‐FAM or NPs+siRNA‐FAM at the indicated N/P ratio for 48 h (scale bar = 100 µm); K) The fluorescent visualization of siRNA‐FAM and lysosome localization in RBE cells after incubation with RBEM@NPs+siRNA‐FAM for 2 h (scale bar = 10 µm) (The blue puncta represented areas stained with Hoechst 33 342, the red puncta signified lysosomes, the green puncta indicated siRNA‐FAM, and the yellow puncta denoted the colocalization of lysosomes and siRNA‐FAM); L,M) The results of proliferation (The time points for the assay were chosen at 0, 1, 2, 3, and 4 days after treatment) and invasion assays (transwell, scale bar = 50 µm) (The time points for the assay were chosen at 48 h after treatment) of ICC cells incubated with Control, HIBEpiCM@NPs+siNC, HIBEpiCM@NPs+si‐ABHD11‐AS1, ICCM@NPs+siNC, and ICCM@NPs+si‐ABHD11‐AS1 (*n* = 3); N,O) The results of qRT‐PCR and western blot assays of ICC cells incubated with Control, HIBEpiCM@NPs+siNC, HIBEpiCM@NPs+si‐ABHD11‐AS1, ICCM@NPs+siNC, and ICCM@NPs+si‐ABHD11‐AS1 (The time points for the assay were chosen at 48 h after treatment) (*n* = 3). The continuous variables of normal distribution were represented as the mean ± standard error of the mean. The ANOVA test was employed for comparing data of multiple groups (F,L–N). *P‐*value < 0.05 was considered statistically significant. *p < 0.05, **p < 0.01, ***p < 0.001.

We have recently developed a novel reactive oxygen species (ROS)‐responsive cationic polymer, CBP5, and used it to encapsulate siRNA for efficient delivery. The compounds 1 and 2 required for synthesis of CBP5 were synthesized in accordance with the methods described in previous reports.^[^ ^41^, ^42^ ^]^ The specific details of the process used to synthesize CBP5 can be found in our patent application (number 202311631799X, filed on November 30, 2023) and in the Experimental Section. The route for synthesis of CBP5 was shown in Figure S5A (Supporting Information). We have also used cancer cell membrane coating nanotechnology to achieve targeted delivery of nanoparticles (NPs) + siRNA specifically to ICC cells. This innovative approach endows NPs with enhanced biocompatibility and reduced immunogenicity, allowing them to evade immune clearance, prolonging their circulation time, and facilitating targeted delivery to cancer cells through the recognition mechanism of membrane proteins, which provides a valuable method for addressing the challenges faced by traditional nanomedicine in the treatment of cancer. We extracted the cell membrane (HIBEpiCM, RBEM, and HuCCT1M) from a human intrahepatic biliary epithelial cell line (HIBEpiC) and two ICC cell lines (RBE and HuCCT1) to package CBP5 + siRNA for targeted delivery (**Figure** **7**A). Western blot assays conducted to detect the protein levels of 3‐phosphoglyceraldehyde dehydrogenase, β‐tubulin, and Na‐K‐ATPase in cytoplasm, cell membrane, and whole cell samples confirmed successful isolation of the cell membrane (Figure 7B). Next, we performed protein mass spectrometry analysis (with label‐free quantification) to further determine the composition of the extracted cell membrane (HIBEpiCM, RBEM, and HuCCT1M) and compared their differences. The results revealed 318 proteins that overlapped between the differential proteins of HIBEpiCM versus RBEM (*n* = 733) and the differential proteins of HIBEpiCM versus HuCCT1M (*n* = 724) (low expression in HIBEpiCM). Reactome enrichment analysis of these 318 overlapped proteins indicated that compared to HIBEpiCM, ICCM had a higher abundance of proteins associated with lipid metabolism (METABOLISM_OF_LIPIDS), as well as proteins related to the protein recognition and transportation of cell membrane and membrane vesicle system (PEROXISOMAL_PROTEIN_IMPORT, PROTEIN_LOCALIZATION, and INTEGRIN_CELL_SURFACE_INTERACTIONS). Taking the differential pathway INTEGRIN_CELL_SURFACE_INTERACTIONS as an example, the pathway involved JAM3, COL16A1, SPP1, COL13A1, ITGA5, ITGB3, COL18A1, and TNC proteins, which participate in cell recognition, adhesion, and interaction processes (Figure S4A–C, Supporting Information; *n* = 3). Previous studies have shown that distant metastatic tissues could take up exosomes derived from primary cancers containing integrin‐related proteins and that these proteins play important roles in recognition of cancer cells and progression of cancer.^[^ ^43^ ^]^ Subsequently, we used western blotting assays to further prove the reliability of the mass spectrometry findings, specifically demonstrating that the INTEGRIN_CELL_SURFACE_INTERACTIONS pathway, led by JAM3, COL16A1, and SPP1, were indeed highly expressed on the membranes of ICC (Figure S4D, Supporting Information), which suggested that the synergistic effect of the above‐mentioned proteins helped to achieve homologous targeting by the cancer cell membrane delivery system. The particle size and zeta potential of NPs + siRNA, RBEM, and RBEM@NPs + siRNA were shown in Figure 7C–G (*n* = 3).

CBP5 could release siRNA in response to ROS and enhance the efficiency of siRNA transfection. The results of exploration of the role of CBP5 in releasing siRNA in response to ROS were as follows. Consistent with conclusions from published articles (Compared to normal cells, the ROS levels are significantly elevated in cancer cells^[^ ^44^, ^45^ ^]^), our flow cytometry assays showed that ICC cells exhibited ROS of higher levels compared to primary cells from adjacent tissues (Figure 7H; *n* = 3). Furthermore, DNA gel electrophoresis showed that hydrogen peroxide significantly promoted the escape of nucleic acids from NPs + siRNA (Figure 7I). Therefore, we inferred that NPs + siRNA were more prone to releasing siRNA in ICC cells because ROS levels were higher in these cells than control cells. The results of exploring the ability of CBP5 to enhance the efficiency of siRNA transfection were as follows. Compared with the use of Lipo3000 or construction of NPs+siRNA‐FAM with other N/P ratios, NPs + siRNA‐FAM showed relatively higher transfection efficiency when the N/P ratio was ≥20 (Figure 7J; Figure S5B, Supporting Information). To balance cell toxicity and transfection efficiency, an N/P ratio of 20 was chosen for subsequent experiments. qRT‐PCR and western blot assays showed that the transfection efficiency of NPs+siABHD11‐AS1 was better than that of Lipo3000 (Figure S5C,D, Supporting Information). When co‐cultured with lysosome (LysoTracker Red)‐labeled RBE cells, all groups showed red puncta representing lysosomes. However, only the NPs+siRNA‐FAM and RBEM@NPs+siRNA‐FAM groups displayed green puncta representing transfected siRNA‐FAM, while the control and bare siRNA‐FAM groups showed no green puncta. Furthermore, compared with the NPs+siRNA‐FAM group, the RBEM@NPs+siRNA‐FAM group had fewer overlapping areas of red and green puncta (Figure 7K). These findings demonstrated that NPs significantly enhanced the transfection efficiency of siRNA‐FAM and that application of RBEM enabled NPs+siRNA‐FAM to escape efficiently from lysosomes.

CCK8 and invasion assays indicated that the proliferation and invasion ability of ICC cells was significantly suppressed in the ICCM@NPs + siABHD11‐AS1 group when compared with the other groups (Figure 7L,M; *n* = 3). qRT‐PCR and western blot assays simultaneously demonstrated that the ICCM@NPs+siABHD11‐AS1 group was best able to inhibit expression of ABHD11‐AS1 in ICC cells (Figure 7N,O; *n* = 3).

### 2.8 Construction of a Synergistic Anticancer Strategy Targeting ECM Stiffness by Integrating ECM Softening and Blocking Intracellular Mechanical Signal Transduction

**Figure 8:**
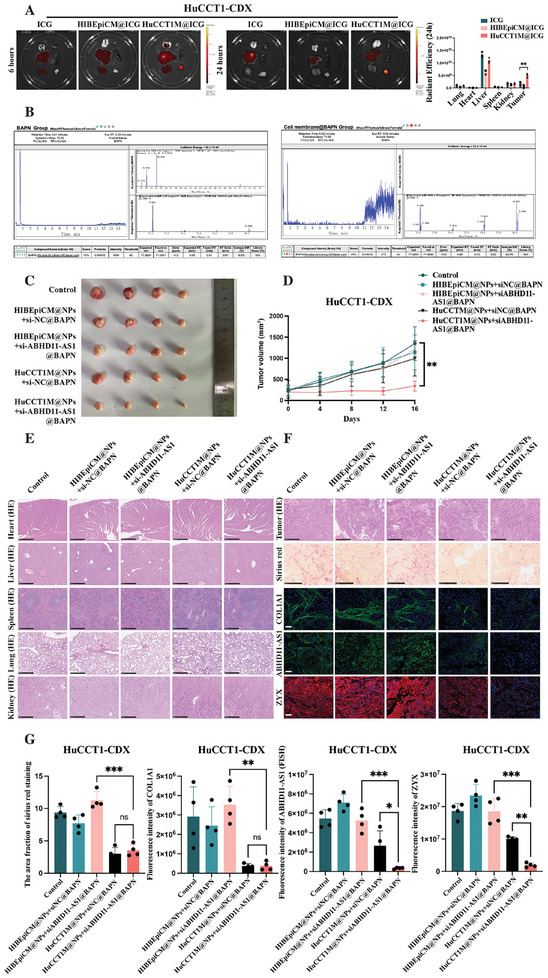
The construction of the synergistic anticancer strategy targeting ECM stiffness by integrating ECM softening and blocking intracellular mechanical signal transduction. A) The exploration of the in vivo targeting ability of cancer cell membrane coating nanotechnology for ICC using cell line‐derived xenograft (CDX) model (The time points for the assay were chosen at 6 and 24 h after treatment) (*n* = 3); B) The encapsulation effect of BAPN using cancer cell membrane coating nanotechnology; C) The comparison of cancer size of five groups (including the Control, HIBEpiCM@NPs+siNC@BAPN, HIBEpiCM@NPs+si‐ABHD11‐AS1@BAPN, HuCCT1M@NPs+siNC@BAPN, and HuCCT1M@NPs+si‐ABHD11‐AS1@BAPN groups) at the end of the experiment (At the 16 days after treatment) (*n* = 4); D) The growth curve of HuCCT1‐CDX in vivo measured every four days (The time points for the assay were chosen at 0, 4, 8, 12, and 16 days after treatment) (*n* = 4); E) The representative images of HE staining depicting the heart, liver, spleen, lung, and kidney in each group (scale bar = 500 µm); F) The representative images of HE staining depicting the tumor in each group (scale bar = 500 µm); The collagen content detected by sirius red staining (scale bar = 500 µm) and immunofluorescence of COL1A1 (scale bar = 50 µm) in each group; The expression of ABHD11‐AS1 detected by FISH (scale bar = 50 µm) in each group; The expression of ZYX detected by immunofluorescence (scale bar = 50 µm) in each group; G) Fluorescence quantification of Figure 8F (*n* = 4). The continuous variables of normal distribution were represented as the mean ± standard error of the mean. The ANOVA test was employed for comparing data of multiple groups (A,D,G). *P*‐value < 0.05 was considered statistically significant. *p < 0.05, **p < 0.01, ***p < 0.001.

After confirming the highly selective and efficient transfection effect of ICCM@NPs + siABHD11‐AS1 in vitro, we conducted further investigations to explore the in vivo targeting ability of cancer cell membrane coating nanotechnology for ICC using the cell line‐derived xenograft (CDX) model. First, we established three HuCCT1‐CDX groups (6 mice per group) and injected ICG solution, ICG‐labeled HIBEpiCM@ICG, or HuCCT1M@ICG into the tail vein of each mouse, respectively. We then detected the in vivo distribution of ICG or ICG‐labeled cell membrane at 6 and 24 h after injection. The results of the HuCCT1‐CDX model showed that compared to the control group, HuCCT1M@ICG was more prone to accumulate in the HuCCT1‐CDX. In summary, in our ICC‐CDX model, use of cell membrane from the homologous cancer cell line to encapsulate NPs achieved excellent homologous targeting outcomes (**Figure** **8**A).

The overall aim of this study was to develop a synergistic strategy for suppression of cancer by combining the degradation of ECM or the reduction of ECM cross‐linking, along with blocking the intracellular response of cancer cells to mechanical signals from the ECM. Therefore, we incorporated BAPN into our anticancer strategy system to soften the ECM. We used liquid chromatography–mass spectrometry to detect the encapsulation effect of BAPN using cancer cell membrane coating nanotechnology. We found that even after removing the non‐encapsulated BAPN, the ICC cell membrane still contained a certain amount of BAPN (the BAPN score of 1.25 mg ml^−1^ BAPN solution was 75%, and the BAPN score of the mixture of 2.5 mg ml^−1^ BAPN solution and the equal volume of ICC cell membrane was 29%) (Figure 8B).

The relevant animal experiments in this study demonstrated that following a 16‐day period of tail vein injections administered, the treatment outcomes achieved by the HuCCT1M@NPs + siABHD11‐AS1@BAPN anticancer strategy were superior to those achieved in the other groups. From the macroscopic perspective, the HuCCT1‐CDX tumor size was significantly smaller in the HuCCT1M@NPs + siABHD11‐AS1@BAPN group than in the other groups (Figure 8C,D; *n* = 4). From the microscopic perspective, sirius red and immunofluorescent staining of COL1A1 showed that the collagen content was significantly lower in the HuCCT1M@NPs + siABHD11‐AS1@BAPN and HuCCT1M@NPs + siNC@BAPN groups than in the other groups. However, FISH assays showed that inhibition of ABHD11‐AS1 expression in the HuCCT1‐CDX model was most effective in the HuCCT1M@NPs+siABHD11‐AS1@BAPN group. Immunofluorescent staining also showed that protein levels of ZXY were significantly lower in the HuCCT1M@NPs+siABHD11‐AS1@BAPN group than in the other groups. Hematoxylin‐eosin staining showed that the treatment methods used did not cause significant damage to the heart, liver, spleen, lungs, or kidneys in any of the groups. During the course of experimental timeline, there was no significant difference in bodyweight change among the five groups (Figure S5E, Supporting Information; *n* = 4). In summary, the synergistic anticancer strategy, HuCCT1M@NPs+siABHD11‐AS1@BAPN, which targeted ECM stiffness by integrating ECM softening and blockade of intracellular mechanical signal transduction, exhibited excellent biosafety and efficacy in ICC (Figure 8E,F; *n* = 4).

## 3 Discussion

Cancer is a complex systemic disease characterized by intricate interactions among cancer cells, the ECM, and various types of cells within the tumor microenvironment. The ECM, as a significant component of cancer, plays multiple roles, including providing mechanical support, modulating the microenvironment, and acting as a source of signaling molecules.^[^ ^2^, ^4^ ^]^ Moreover, dysregulation of the ECM is a prominent characteristic of cancer, contributing to its progression and affecting tumor behavior.^[^ ^46^ ^]^ Throughout tumorigenesis, the interaction between cancer cells and the tumor microenvironment increases ECM stiffness, triggering abnormal mechanotransduction and facilitating further malignant transformation.^[^ ^46^ ^]^ Stiffening of the ECM relies heavily on excessive deposition and cross‐linking within the ECM, so it can be prevented or reversed by degrading the ECM and reducing its cross‐linking. Hyaluronidase and LOX inhibitors are often used to target modulation of the ECM. A recent study demonstrated that degradation of hyaluronan in vivo using a clinical dose of hyaluronidase suppressed tumorigenesis of mesenchymal colorectal cancer and its metastasis to the liver.^[^ ^47^ ^]^ BAPN is a specific LOX inhibitor, and its mechanism of action involves competitive binding to the active site of the LOX enzyme, preventing it from oxidizing amino acid residues. This action decreases the cross‐linking of collagen fibers, thereby affecting the structure and mechanical properties of the ECM.^[^ ^23^ ^]^ Although LOX inhibitors and hyaluronidase could be adopted to prevent the progression of pathology caused by matrix cross‐linking, it has some limitations that have been impeding its clinical application, including toxicity and the fact that softening or degradation of the ECM also eliminates obstacles to invasion of cancer.^[^ ^25^, ^26^ ^]^ The mechanical constraints of type I collagen can counteract the cancer‐promoting effects of cancer‐associated fibroblasts, which suggests that blindly degrading the ECM may have unintended consequences and that preserving type I collagen while targeting cancer‐associated fibroblasts may be more advantageous.^[^ ^27^ ^]^ To overcome these limitations, we proposed integrating the degradation of ECM or reduction of ECM cross‐linking, along with blocking the intracellular response of cancer cells to mechanical signals from the ECM.

Specifically, our novel delivery system consists of three components, namely NPs (CBP5) + siRNA, BAPN, and cell membrane. NPs (CBP5) were first developed by our team, and we found that they could improve the transfection efficiency of siRNA as well as serve as a ROS‐responsive charge‐switchable cationic polymer. Targeted RNA delivery faces persistent challenges because of the limited targeting ability of specific cells and the destructive acidic environment of lysosomes, which hamper effective application of RNAi technology in the treatment of cancer.^[^ ^48^ ^]^ Therefore, it is necessary to develop a spatially selective RNAi delivery system that specifically targets cancer cells. In recent years, researchers have proposed the use of cell membrane coating nanotechnology as a strategy to enhance the interaction between NPs and the human body. Our team has also previously made advances in application of cell membrane coating nanotechnology for the treatment of osteoarthritis.^[^ ^49^ ^]^ NPs prepared using this strategy inherit specific biological functions from the source cells (e.g., cancer cells), allowing them to integrate the superiority of various proteins and molecules on the cell membrane. Cancer cell membrane coating nanotechnology confers NPs with better biocompatibility and lower immunogenicity, enabling evasion of immune clearance, prolonging circulation time, and facilitating targeted delivery to cancer cells through the ligand–receptor recognition mechanism of membrane proteins, thus providing a potential solution to the challenges faced by traditional nanomedicine in cancer treatment.^[^ ^50^, ^51^ ^]^ Furthermore, to achieve a synergistic anticancer strategy that combines degradation of the ECM or reduction of ECM cross‐linking along with inhibition of intracellular responses of cancer cells to mechanical signals from the ECM, we incorporated BAPN, a specific LOX inhibitor. As mentioned above, BAPN has the ability to reduce the cross‐linking of collagen fibers, thereby influencing the structure and mechanical properties of the ECM.^[^ ^23^ ^]^ This integrated approach could exploit the distinctive strengths of its constituent elements and foster robust mutual complementarity. On the one hand, tackling tumoral ECM could improve the delivery of lipid nanoparticles in patients with cancer. For example, Daniel J. Siegwart et al. demonstrated that inhibiting FAK led to a reduction of the contractile force and membrane tension properties of tumor cells and ECM stiffness, which significantly facilitated CRISPR gene editing in tumor cells by increasing the endocytosis and tumor penetration of lipid nanoparticles.^[^ ^52^ ^]^ On the other hand, NPs could regulate the intracellular response of cancer cells to mechanical signals from the ECM and influence cancer progression, thereby diminishing the likelihood of tumor dissemination resulting from degradation of the ECM.

In this study, the immunofluorescence of tissue microarrays of primary liver cancers (91 ICC cases and 90 HCC cases) and bioinformatics analysis of TCGA mRNA sequencing data, including TCGA‐CHOL (*n*[T] = 36, *n*[N] = 9) and TCGA‐LIHC (*n*[T] = 369, *n*[N] = 50) revealed that collagen‐related molecules influenced the long‐term prognosis of primary liver cancers and that higher expression levels of these molecules were associated with a worse long‐term prognosis. The ECM is significantly stiffer in ICC than in HCC, and ICC has a higher degree of malignant behavior and a poorer long‐term prognosis (Figure 1). However, there is currently a dearth of published articles exploring the relationship between ECM stiffness and the onset and progression of ICC. Therefore, we used ICC to explore further the specific mechanisms underlying the impact of ECM stiffness on progression of cancer and the effects of application of integrated anticancer strategies targeting ECM stiffness with the aim of extending the applicability of this strategy to various clinical scenarios.

We used the YAP1/ABHD11‐AS1/STAU2/ZYX/p‐YAP1 signaling pathway as the entry point to explore the specific mechanisms involved in conduction of mechanical signals from the ECM in ICC cells and their impact on progression of cancer. Non‐coding RNAs, which cannot encode proteins, comprise more than 90% of the RNAs made from the human genome,^[^ ^53^ ^]^ while lncRNAs (defined as ncRNA transcripts longer than 200 nucleotides^[^ ^54^ ^]^), account for 80% of non‐coding RNAs and play important roles in various cellular functions by participating at multiple regulatory levels (i.e., transcription, post‐transcription, translation, post‐translation, and epigenetics).^[^ ^55^ ^]^ Therefore, we first screened for functional lncRNAs related to ECM stiffness in ICC using transcriptome sequencing, an siRNA library (containing 237 siRNAs targeting 79 lncRNAs that are differentially expressed between ICC and adjacent tissues) functional experiments, and ECM stiffness‐related lncRNA screening experiments. Ultimately, we anchored to the novel transcript (T2) of ABHD11‐AS1. ABHD11‐AS1 is a lncRNA with carcinogenic potential that has been attracting attention in recent years because of its important role in progression of cancer.^[^ ^56^, ^57^, ^58^ ^]^ But there have been no articles discussing the relationship between ABHD11‐AS1 and primary liver cancers or ECM stiffness. Our study demonstrated that ECM stiffness and the stiffness‐related transcription co‐activator YAP1 could positively regulate transcription of ABHD11‐AS1 in the upstream direction. In the downstream direction, in the cytoplasm, ABHD11‐AS1 could interact with STAU2, an RNA‐binding protein, to stabilize ZYX mRNA, thereby preventing degradation of the latter. Our findings regarding the functionality of ZYX were consistent with previous findings.^[^ ^38^, ^59^ ^]^ And this study further proposed that under the influence of ABHD11‐AS1, ZYX affected phosphorylation modification and nuclear localization of YAP1, thereby influencing the response of ICC cells to mechanical signals from the ECM, as well as the proliferation, invasion, and other malignant phenotypes of ICC cells.

To minimize potential damage to normal cells, we chose the functional molecule ABHD11‐AS1, which was differentially expressed in ICC and adjacent tissues and responded to mechanical signals from the ECM, as the intracellular regulatory target. In accordance with a previous study by our team,^[^ ^49^ ^]^ to achieve targeted delivery to ICC cells, we extracted the ICC cell membrane to coat our team's newly developed NPs (CBP5), si‐ABHD11‐AS1, along with BAPN (forming the ICCM@NPs+si‐ABHD11‐AS1@BAPN treatment system). Compared with the control HIBEpiCM, ICCM showed elevated expression of proteins associated with the INTEGRIN_CELL_SURFACE_INTERACTIONS pathway, which participates in cell recognition, adhesion, and interaction processes. Notably, previous research has demonstrated that exosomes derived from primary cancers that contain integrin‐related proteins can be taken up by distant metastatic tissues, and integrin‐related proteins play important roles in recognition of cancer cells and progression of cancer.^[^ ^43^ ^]^ The relevant animal experiments in this study demonstrated that the treatment outcomes were better in the HuCCT1M@NPs+siABHD11‐AS1@BAPN group than in the other groups, that is, the HuCCT1‐CDX tumor size was significantly reduced in the HuCCT1M@NPs+siABHD11‐AS1@BAPN group. Notably, HuCCT1M@NPs+siABHD11‐AS1@BAPN decreased the collagen content of HuCCT1‐CDX tissues, effectively inhibited the intracellular response of ICC cells to mechanical signals from the ECM, and had excellent biosafety and efficacy.

## 4 Conclusion

The findings of this study demonstrated that ECM stiffness could affect the phenotype of primary liver cancers and that the YAP1/ABHD11‐AS1/STAU2/ZYX/p‐YAP1 signaling pathway was an entry point that could be used to explore the specific mechanisms of mechanical signal conduction from the ECM in ICC cells and their impact on progression of cancer. Moreover, we also constructed a synergistic anticancer strategy (ICCM@NPs+siABHD11‐AS1@BAPN) that targeted ECM stiffness by integrating softening of the ECM and blocking of intracellular mechanical signal transduction in ICC. Finally, we demonstrated that this novel approach had better therapeutic effects, reduced the frequency of drug‐related adverse events, and could provide promising insights in terms of treatment for cancers characterized by a stiff ECM.

## 5 Experimental Section

### Statistical Analysis

The continuous variables of normal distribution were represented as the mean ± standard error of the mean; the continuous variables of skew distribution were represented by the median (range); and the count data was represented by the number of cases (percentage). Student's *t*‐test was employed to compare continuous variables following normal distribution, while the Mann–Whitney *U*‐test was used for non‐normally distributed continuous variables. The chi‐square test, or Fisher's exact test, was conducted for categorical variables. The analysis of variance test was employed for comparing data of multiple groups. Survival analysis was performed using the Kaplan–Meier method, and survival curves were simultaneously plotted. The log‐rank test was used to compare the different groups' overall survival. Sample size (*n*) for each statistical analysis was indicated in the corresponding result and figure legend sections. *P*‐value <0.05 was considered statistically significant. All statistical analyses were conducted using R 4.1.2 and GraphPad Prism 9 software.

### Ethical Approval

This study involving human participants was reviewed and approved by Ethics Committee in Clinical Research of Sir Run Run Shaw Hospital, School of Medicine, Zhejiang University (NO. 20230211‐127). The data used in this article were all items that must be checked according to medical standards during the hospitalization, and collected retrospectively when designing the study, without adding any additional medical examination or test outside the normal diagnosis and treatment procedures.

All animal experiments were carried out according to the regulations proposed by the Association for the Assessment and Accreditation of Laboratory Animal Care and the Institutional Animal Care and Use Committee Guidelines and approved by the Ethics Review Committee for Animal Welfare in Experiments of Sir Run Run Shaw Hospital, School of Medicine, Zhejiang University (SRRSH202302163).

## Conflict of Interest

The authors declare no conflict of interest.

## Author Contributions

Z.F.S., L.Y.T., Y.L.W., and Y.W.Z. contributed equally to this study. X.L., J.J.X., L.Q.S.G., Z.F.S., and L.Y.T. were responsible for the conception, design, and writing of the article. Z.F.S., L.Y.T., Y.L.W., and Y.W.Z. were responsible for the data processing and analysis. Z.F.S., Y.L.W., L.Y.T., Y.W.Z., H.Y.P., Y.J.L., S.J., J.H.Z., Y.L., K.N.L., and S.H.L. were responsible for collecting the original data. Z.F.S., X.L., J.W.C., and Y.F.T. were responsible for reviewing and guiding the revision of the paper. All authors contributed to the article and approved the submitted version.

## Supporting information



Supporting Information

Supplemental Table 1

## Data Availability

The data that support the findings of this study are available in the supplementary material of this article.
