# Supplementary material for: Synergistic Anticancer Strategy Targeting ECM Stiffness: Integration of Matrix Softening and Mechanical Signal Transduction Blockade in Primary Liver Cancers
Source: Adv Sci (Weinh). 2024 Dec 20;12(6):2403040. doi: 10.1002/advs.202403040 (PMC11809367; doi:10.1002/advs.202403040)
Supplement: Supplementary file 1 — Supporting Information [file ADVS-12-2403040-s001.docx]

**Experimental Section**

**Datasets and Samples**

90 tumor tissues of HCC patients suffering hepatectomy were collected from Zhejiang University, School of Medicine, Sir Run Run Shaw Hospital, Hangzhou, China, from April 2014 to November 2015 and manufactured into tissue microarrays for immunofluorescence. And another 4 pairs of fresh tumor tissues and adjacent tissues from HCC patients in November 2022 were used for atomic force microscope (AFM) analysis, collagen detection, and immunofluorescence.

91 tumor tissues of ICC patients suffering hepatectomy were collected from Zhejiang University, School of Medicine, Sir Run Run Shaw Hospital, Hangzhou, China, from January 2014 to December 2019 and manufactured into tissue microarrays for immunofluorescence. Another 19 pairs of tumor tissues and adjacent tissues from ICC patients from September 2016 to May 2018 were performed bulk RNA-Sequencing profiling. And another 4 pairs of fresh tumor tissues and adjacent tissues from ICC patients in November 2022 were used for AFM analysis, collagen detection, and immunofluorescence.

The process of AFM sample preparation was as follows. In the sterile operating room, we divided the required specimens into 1cm*1cm*1cm tissue blocks, put them into sterile and dry plastic bags. And then the plastic bags was put into a low-temperature container with ice cubes or ice packs for transportation. Finally, the tissue blocks were placed on the stage of the atomic force microscope for detection of tissue stiffness.

The TCGA mRNA sequencing data were downloaded as external validation, including TCGA-CHOL (T=36, N=9) and TCGA-LIHC (T=369, N=50). The specific information for all samples was shown in Table 1. The study was approved by the Ethics Committee of SRRSH.

**Identification of lncRNAs Using RNA-Sequencing**

1. Library Preparation and Sequencing: RNA sequencing (RNA-seq) libraries were prepared using RNA-seq library preparation kit with ribosomal RNA (rRNA) depletion or poly(A) selection to enrich for non-ribosomal transcripts. The libraries were sequenced on the illumina PE150 platform to generate paired-end reads. The work of library preparation and sequencing was completed by Novogene company (China).

2) Quality Control: Raw sequencing reads were evaluated for quality using FastQC. Adapters and low-quality bases were trimmed using Trimmomatic^1^, ensuring that only high-quality reads were retained for downstream analysis.

3) Alignment: Trimmed reads were aligned to the human reference genome (hg38) using the splice-aware aligner HISAT2^2^. The alignment process was configured with default settings to allow for the identification of known splice junctions and accurate mapping of reads to the transcriptome.

4) Assembly and Quantification: Aligned reads were assembled into transcripts using StringTie^3^. It reconstructed full-length isoforms from RNA-seq data, enabling the identification of both known and novel transcripts. Aligned reads were quantified to estimate transcript expression levels using HTSeq^4^. Expression levels were normalized to account for sequencing depth and gene length, resulting in transcripts per million (TPM).

5) Identification of known lncRNAs: After transcriptome sequencing and data preprocessing, the lncRNAs were identified and annotated with known sequences using lncRNA databases, including GENCODE, NONCODE, and LncRNADisease ^5-7^.

6) Validation: Selected lncRNA candidates were validated using quantitative reverse transcription PCR (qRT-PCR) to confirm their expression patterns. The full-length lncRNA was obtained using the 5'RACE and 3'RACE kits (SMARTer® RACE 5'/3' Kit Protocol) (Takara Bio, USA), and its specific sequence was further analyzed.

**Cell culture and transfection experiments**

Tested negative for mycoplasma contamination, the human ICC cell lines (RBE, HuCCT1, 9810) were purchased from Meisen cell technology Co. Ltd., Meisen Chinese Type Culture Collection (MeisenCTCC, China) and human intrahepatic biliary epithelial cell line (HIBEpiC) was donated by Dr Shi Jiang from Zhejiang University. The RBE and HuCCT1 cell lines were maintained in Dulbecco modified Eagle medium (DMEM, Gibco, USA) supplemented with 10% fetal bovine serum at 37°C in 5% CO_2_ condition. The 9810 and HIBEpiC cell lines were maintained in RPMI 1640 (Gibco, USA) containing 10% fetal bovine serum at 37°C in 5% CO_2_ condition. According to the manufacturer’s instructions, Lipofectamine 3000 Reagents (Thermo Fisher Scientific, USA) were adopted to achieve the transfection of siRNA (Ribobio, China) and plasmids.

**Invasion assays**

To start, pre-coat the transwell upper chamber (Corning, USA) with diluted Matrigel (Yeasen, China) as needed and incubate it in a constant temperature incubator for 4 hours. Next, follow the method above, digest and centrifuge the treated cells, then resuspend them in serum-free DMEM medium for later use. Afterward, seed approximately 3×10^4^ cells in the upper chamber of a 24-well plate containing Transwell inserts and add 600μL of serum-containing DMEM medium to the lower chamber. Once the cells have invaded for 24-48 hours, gently wash them in the 24-well plate with PBS to remove detached cells, and fix them with 4% paraformaldehyde for 15 minutes. Proceed to stain the cells in the upper and lower chambers with crystal violet staining solution, wash off the excess dye with gentle PBS washing, and lightly wipe the upper chamber to remove the cells, leaving only the invasive cells in the lower chamber. Following that, capture images using a Nikon inverted microscope. Finally, cell counting analysis was performed using Image J software, and the results were compared between different treatment groups.

**CCK-8 assays**

We detected cell proliferation using the Cell Counting Kit (CCK-8) (Yeasen, China). Cells were seeded in the 96-well plate and treated accordingly. Following the experimental purpose, the cells were cultured for a specific period, and then the CCK-8 solution was added at a 10% proportion. The mixture was further incubated for 1-2 hours in a CO_2_ incubator. Next, the absorbance OD value was measured at a wavelength of 450nm using a microplate reader. Complete operation instructions could be found in the user manual of CCK-8 (Yeasen, China).

**EdU assays**

The EdU assay was performed using the Yefluor 594 EdU Imaging Kits (Yeasen, China). Cells were seeded at a density of 10^4^ cells per well in a 96-well plate and treated accordingly. A suitable amount of EdU working solution was prepared and incubated with the cells for 48h. Subsequently, the cells were fixed and permeabilized using neutral polyformaldehyde and glycine. Cell washing was carried out with 3% BSA, followed by permeabilization with 0.5% Triton X-100. EdU detection was performed by preparing the Click-iT reaction mixture and incubating it in each well. And DNA re-staining was performed using Hoechst 33342. Following that, capture images using a fluorescence microscope. Following that, capture images using a fluorescence microscope. Complete operation instructions could be found in the user manual of Yefluor 594 EdU Imaging Kits (Yeasen, China). Additionally, the Image J software was used to determine the total staining intensity, which is the multiplying of the mean staining intensity and the staining area.

**In vitro experiment exploring the effect of ECM stiffness on the phenotype of ICC cells**

In the cell experiments, 6-well CytoSoft® plates (Advanced biomatrix company, USA) with differences in Young's modulus (2 kPa and 16 kPa) (using Type I Collagen as the coating material) were used to simulate a difference in ECM stiffness. After incubating on 6-well CytoSoft® plates of 2kpa and 16kpa for 48 hours, we collected cells from each group and count them. After ensuring that the initial number of cells per well from different groups of the same cell line was the same, we conducted subsequent phenotype assays.

**Quantitative real-time PCR (qRT-PCR) and western blot assays**

The total RNA was extracted using the RNA Extraction Kit (Yishan, China), following the manufacturer's instructions. Subsequently, 1-2 µg of total RNA was reverse transcribed into complementary DNA using the Hifair II 1st Strand cDNA Synthesis Kit (Yeasen, China). For quantitative real-time PCR (qRT-PCR), SYBR Green Master Mix (Yeasen, China) was utilized, and the detection was conducted using the LightCycler 480 instrument (Roche, Switzerland). The relative expression of RNA was normalized using glyceraldehyde-3-phosphate dehydrogenase (GAPDH). The primers employed in this study were listed in Table S2.

Containing a protease inhibitor (Thermo Fisher Scientific, USA) and a phosphatase inhibitor, the RIPA lysis buffer (Beyotime, China) was used for total protein extraction. The extracted proteins were separated on a 10–12% SDS/PAGE gel. Subsequently, the proteins were transferred onto a PVDF membrane for protein transmembrane. Following overnight incubation of the transferred membrane with the corresponding primary antibody at 4°C and washing with a 10% TBST solution, the membrane was incubated with the corresponding secondary antibody for 1 hour at room temperature. Finally, after washing with a 10% TBST solution, the antigen-antibody complex on the membrane was detected using an enhanced chemiluminescence reagent (Fdbio Science, China). The antibodies used in this study are listed in Table S3.

**Actinomycin D assays**

The cells were first seeded into a 6-well plate. The following day, the cells were transfected with si-NC and si-RNA of the molecule under investigation, and the medium was changed after 8 hours. 24 hours after the medium change, actinomycin D (MCE, USA) was added to each well. At 0h, 3h, 6h, and 9h (or other time gradients), a well was respectively selected to obtain cell precipitation. RNA was then extracted from the cell precipitation for subsequent qRT-PCR.

**Chromatin isolation by RNA purification assays**

In order to explore the binding states of upstream RNA to the promoter region of downstream target molecules, we conducted a chromatin isolation by RNA purification (ChIRP) assays using the ChIRP Assay Kit (Ribobio, China). The specific steps were as follows: 1) Collecting cells to be used for the ChIRP assay; 2) Crosslinking the cells from step 1 with glutaraldehyde to preserve the RNA-chromatin interactions and performing cell precipitation; 3) Dissolving the crosslinked cells to prepare a cell lysate; 4) Sonication of the crosslinked cell lysate to shear DNA; 5) Hybridizing biotinylated DNA probes to RNA and separating the bound chromatin; 6) Extracting DNA fragments from the ChIRP samples, identifying and quantifying them using qRT-PCR. Complete operation instructions could be found in the user manual of Yefluor 594 EdU Imaging Kits (Yeasen, China).

**Immunohistochemistry, immunofluorescence, and fluorescence in situ hybridization (FISH) assays**

The tissues were fixed with 4% paraformaldehyde and embedded in paraffin. Then, they were cut into 3-μm-thick sections and stained with appropriate primary antibodies for immunohistochemistry or immunofluorescence. The sections were incubated overnight at 4 °C with the primary antibodies. After washing with PBS as recommended, the sections for immunohistochemistry were treated with the GTvision immunohistochemistry kit following the manufacturer's instructions, and the sections for immunofluorescence were treated with the corresponding fluorescent secondary antibodies. The FISH assay was conducted according to the manufacturer's instructions (GenePharma, China). Following that, capture images using a fluorescence microscope. Additionally, the Image J software was used to determine the total staining intensity, which is the multiplying of the mean staining intensity and the staining area.

**Chromatin immunoprecipitation (ChIP) assays**

We performed ChIP assays using the ChIP Assay Kit (Beyotime, China) to study the interaction between proteins and chromatin (e.g., YAP1 and promoter regions of ABHD11-AS1). The experimental procedure included the following steps:1) cross-linking and fixation of cells or tissues to preserve the protein-chromatin interaction; 2) lysis of cells or tissues to release chromatin; 3) immunoprecipitation of the target protein bound to chromatin using specific antibodies; 4) washing of the precipitated complex to remove nonspecific binding molecules; 5) reverse cross-linking to restore the original chromatin structure; 6) DNA purification to obtain DNA fragments bound to the target protein; 7) qRT-PCR assays of the DNA fragments to identify and analyze DNA sequences interacting with the target protein. Complete operation instructions could be found in the user manual of ChIP Assay Kit (Beyotime, China).

**Dual-luciferase reporter assays**

In order to investigate whether the binding of YAP1 and the promoter regions of ABHD11-AS1 could facilitate the transcription of the latter, we conducted a dual-luciferase reporter assay. Firstly, we obtained the promoter region fragment of ABHD11-AS1 and its corresponding mutant fragment through PCR. Subsequently, the ABHD11-AS1 promoter region and its corresponding mutant fragment were integrated into the pGL3-basic plasmid using recombinant enzyme-directed cloning technology. The pGL3-basic plasmid containing the PCR fragment and the pRL-TK plasmid containing the renilla luciferase gene were co-transfected into RBE cells. After one week of cultivation, si-NC and si-YAP1 were separately added, and the cells were further cultivated for 48 hours before conducting the dual-luciferase reporter assay (Yeasen, China).

**Biotinylated RNA pull-down assays and RNA immunoprecipitation**

We utilized the RNAmax-T7 Biotin Labeling Transcription Kit (Ribobio, China) to transcribe RNA in vitro (including sense and antisense strands of ABHD11-AS1-T1, sense and antisense strands of ABHD11-AS1-T2, and sense and antisense strands of ZYX mRNA). For the RNA pull-down assays, streptavidin magnetic beads (Thermo Fisher Scientific, USA) were employed. The beads were first washed with RNase-free lysis buffer and then blocked with BSA. The blocked beads were subsequently incubated with the biotinylated RNA sense strand, along with the corresponding antisense control and cell lysate containing a minimum of 1*10^7^ cells, at 4°C for 1 hour. For RNA detection, TRIzol reagent was used to extract RNA from the RNA pull-down products, followed by qRT-PCR. For protein detection, protein extraction was performed on the RNA pull-down products, followed by either protein mass spectrometry or western blot assays. Complete operation instructions could be found in the user manual of RNAmax-T7 Biotin Labeling Transcription Kit (Ribobio, China).

We pre-collected and processed (e.g., transfected with siRNA) an appropriate amount of cell precipitation, and they were subjected to RNA immunoprecipitation using Protein A/G Agarose Beads (Santa Cruz, USA) according to the protocol. At 4°C overnight, lysis buffer-rewashed Protein A/G Agarose Beads were incubated with cell lysates and 2ug of corresponding antibodies. Then, the complex was centrifugally washed three times. For RNA detection, TRIzol reagent was used to extract RNA from the RIP products, followed by qRT-PCR. For protein detection, protein extraction was performed on the RIP products, followed by western blot assays.

**Establishing PDX and CDX models and the metastasis model of ICC cells**

According to the previous study of our team^8^, For the construction of patient-derived xenografts (PDX), the tumor tissue of a 59-year-old male ICC patient hospitalized at Sir Run Run Shaw Hospital, School of Medicine, Zhejiang University, was first implanted into the subcutaneous region of 4-week-old male NOD/SCID mice. After 3 months, the NOD/SCID tumor-bearing mice were euthanized, and the tumor tissue with a size of 1*1*1 mm^3^ were then implanted into the subcutaneous region of 4-week-old male BALB/c nude mice. After 3-4 weeks, when the tumors of nude mice grew to 0.4 cm in length, the nude mice were randomized into different groups to receive corresponding processing strategies.

For the construction of cell-derived xenografts (CDX), 5 × 10^6^ ICC cells suspended in 100 μL PBS were injected into the subcutaneous region of 4-week-old male NOD/SCID mice. After 1-2 months, the tumor-bearing NOD/SCID mice were euthanized, and the tumor tissue with a size of 1*1*1 mm^3^ were then implanted into the subcutaneous region of 4-week-old male BALB/c nude mice. After 3-4 weeks, when the tumors of nude mice grew to 0.4 cm in length, the nude mice were randomized into different groups to receive corresponding processing strategies.

For the construction of the metastasis model of ICC cells, 5 × 10^6^ transfected sh-NC and sh-ABHD11-AS1_003 ICC cells suspended in 100 μL PBS were injected into the tail vein of 4-week-old male BALB/c nude mice. Three months later, the tumor-bearing nude mice were euthanized, and their livers and lungs were dissected to assess the presence of metastasis of ICC cells.

**ROS quantitative assay**

The specific steps we followed to measure the levels of intracellular ROS in the human ICC cell lines (RBE, HuCCT1, 9810) and primary cells from adjacent tissues (primary culture of adjacent tissues from a ICC patient) were as follows:1) Centrifuge the cell suspension, which has been washed with serum-free cell culture medium, at 1000 × g for 5-10 minutes. Collect the cell pellet and resuspend it in the working solution, adjusting the cell concentration to 1×10^5^-1×10^6^/mL; 2) Incubate the cells at 37℃, avoiding light, for 45 minutes. Mix the cells every 3-5 minutes to ensure thorough contact between probes and cells; 3) Centrifuge the cells at 1000 × g for 5-10 minutes. Wash the cells 2-3 times with serum-free cell culture medium to remove any DCFH-DA that did not enter the cells; 4) Resuspend the collected cell pellet in serum-free cell culture medium and detect using a flow cytometer. Complete operation instructions could be found in the user manual of ROS Fluorometric Assay Kit (Elabscience, China).

**Preparation and characterization of NPs**

CBP5 was synthesized based on the invention patent submitted by our team: 202311631799X filed on November 30, 2023 (The synthetic route to CBP5 was illustrated in Figure S5A). Briefly, Compound 1 and Compound 2 were synthesized following the methods described in previous studies^9, 10^. To synthesize CBP5, 2-(4-(Bromomethyl)phenyl)-4,4,5,5-tetramethyl-1,3,2-dioxaborolane (0.98 g, 3.30 mmol) were added to a solution of compound 2 (0.48 g, 0.30 mmol) in acetonitrile (50 mL). The resulting mixture was stirred and refluxed for 24 hours. After cooling to room temperature, the mixture was concentrated by evaporation. Excess ether was then added to the concentrated solution, and the resulting precipitate was collected by filtration. The solid was washed three times with ice ether to remove any residual reactants. The obtained CBP5 (1.20 g, yield 87%) was a white solid, after being dried under vacuum.

According to the previous study of our team^11^, PBS buffer solution was used to dilute siRNA to a concentration of 40 μg/ml. CBP5 was dissolved in PBS buffer solution at various concentrations according to the preset N/P ratios (N/P =10, 15, 20, 25, and 30). Then, equal volumes of siRNA solution were added to the CBP5 solution. The mixture was immediately vortexed for 1 min and then statically incubated for 30 min to obtain polyplexes. The sizes and zeta potentials of the polyplexes were measured by dynamic light scattering (DLS). The encapsulation efficiency of NPs+siRNA with various N/P ratios and ROS responsiveness was evaluated by gel retardation assay. The polyplexes were electrophoresed on a 1% agarose gel at 100 V for 30 min. Gel Red was used in the agarose gel for siRNA detection. The morphology of polyplexes stained with water-soluble phosphotungstic acid was visualized using a transmission electron microscope. For ROS responsiveness assessment, siRNA polyplexes with different N/P ratios were treated with 5 mM H_2_O_2_ at 37°C for 1 hour and then subjected to electrophoresis.

**Isolation of cell membrane**

According to the previous study of our team^11^, RBE, HuCCT1, and HIBEpiC cells were suspended at a density of 5.0 × 10^6^ cells/ml in ice-cold TM buffer [50 mM Tris HCl and 10 mM Magnesium (pH 7.5)] solution and subsequently extruded through a mini-extruder 40 times to disrupt the cells. Sucrose (1 M) was subsequently mixed with the cell homogenate to a final concentration of 0.25 M sucrose, and the mixture was centrifuged at 4°C and 2000g for approximately 10 min. The resulting supernatant was collected via further centrifugation at 3000g for an additional 35 min. The cell membranes were collected and rinsed with ice-cold TM buffer in 0.25 M sucrose for purification. RBEM, HuCCT1M, and HIBEpiCM vesicles were obtained by extruding the purified cell membranes through the same set of porous membranes. A bicinchoninic acid (BCA) protein assay was used to analyze the total protein content in the purified RBEM, HuCCT1M, and HIBEpiCM. The membrane material was stored at −80°C for future study. The membrane protein of RBEM, HuCCT1M, and HIBEpiCM were analyzed by liquid chromatography–tandem mass spectrometry (LC-MS/MS). And the result of LC-MS/MS is listed in Table S6.

**Preparation and characterization of cell membrane@NPs+siRNA**

After isolating the RBEM, HuCCT1M, and HIBEpiCM, the membranes were suspended in PBS buffer solution (0.2 to 1 mg/ml). After ultrasonication and filtration with 0.22μm filter elements, the cell membrane was mixed with an equal volume of prepared NPs+siRNA. The mixture was shaken on a shaker at 70 rpm for half an hour, followed by centrifugation at 800g for 10 minutes to remove the supernatant. The pellet was resuspended in PBS according to the desired volume. The sizes and zeta potentials of the membrane-coated NPs+siRNA were measured by DLS. Morphological examination of the membrane-coated NPs+siRNA was performed using a transmission electron microscope.

**Subcellular distribution analysis**

According to the previous study of our team^11^, to label the lysosomes, the cells were further incubated with LysoTracker Red (Beyotime, China) at a concentration of 200 nM for 15 min after incubation of RBEM@NPs+siRNA for 2 hours, and then the nuclei were stained with two drops of Hoechst 33342 per milliliter of medium for 15 min. The cells were rinsed three times with PBS before observation by confocal fluorescence microscopy.

**In vivo HuCCT1-CDX targeting experiment**

The specific HuCCT1-CDX binding of HuCCT1M was confirmed using an IVIS Spectrum In Vivo Imaging System. ICG-loaded HIBEpiCM or HuCCT1M were prepared as previously described^11^. Mice with HuCCT1-CDX were intravenously injected with ICG, ICG-HIBEpiCM, and ICG-HuCCT1M solutions (10 μl/g) into the tail vein. The biodistribution analysis of ICG was performed with a 710-nm excitation wavelength and a 785-nm filter at 6 hours and 24 hours after injection. Furthermore, the heart, liver, spleen, lung, and kidneys were harvested and analyzed in vitro for the distribution of ICG-HIBEpiCM and ICG-HuCCT1M. Living Image software was used to quantify the fluorescence intensity.

**The construction of the synergistic anticancer strategy targeting ECM stiffness by integrating ECM softening and blocking intracellular mechanical signal transduction**

After isolating the HuCCT1M and HIBEpiCM, the membranes were suspended in PBS buffer solution (0.2 to 1 mg/ml). After ultrasonication and filtration using 0.22μm filter elements, the cell membrane was mixed with 20 mg/ml BAPN and prepared NPs. In the mixture, the volume ratio of cell membrane, 20 mg/ml BAPN, and prepared NPs+siRNA is 1:1:1. The mixture was shaken on a shaker at 70 rpm for half an hour, followed by centrifugation at 800g for 10 minutes to remove the supernatant. The pellet was resuspended in PBS according to the desired volume.

HuCCT1-CDX were implanted into the subcutaneous region of 4-week-old male BALB/c nude mice. After 3-4 weeks, the HuCCT1-CDX models were successfully established in 20 nude mice and these mice were divided into 5 groups. Mice bearing HuCCT1-CDX tumors were intravenously injected with PBS, HIBEpiCM@NPs+siNC@BAPN, HIBEpiCM@NPs+siABHD11-AS1@BAPN, HuCCT1M@NPs+siNC@BAPN, and HuCCT1M@NPs+siABHD11-AS1@BAPN via the tail vein. After another 16 days of treatment, the mice were euthanized. And tumors were harvested and frozen in liquid nitrogen or fixed in 4% formalin immediately.

**Statistical analysis**

The continuous variables of normal distribution were represented as the mean ± standard error of the mean; the continuous variables of skew distribution were represented by the median (range); and the count data was represented by the number of cases (percentage). Student's t-test was employed to compare continuous variables following normal distribution, while the Mann-Whitney U test was used for non-normally distributed continuous variables. The chi-square test, or Fisher's exact test, was conducted for categorical variables. The analysis of variance test was employed for comparing data of multiple groups. Survival analysis was performed using the Kaplan-Meier method, and survival curves were simultaneously plotted. The log-rank test was used to compare the different groups' overall survival. Sample size (n) for each statistical analysis is indicated in the corresponding result and figure legend sections. P value <0.05 was considered statistically significant. All statistical analyses were conducted using R 4.1.2 and GraphPad Prism 9 software.

**References**

1 Anthony M B, Marc L, Bjoern U. Trimmomatic: a flexible trimmer for Illumina sequence data. Bioinformatics 2014; 30 (15).

2 Daehwan K, Ben L, Steven L S. HISAT: a fast spliced aligner with low memory requirements. Nat Methods 2015; 12 (4).

3 Mihaela P, Geo M P, Corina M A et al. StringTie enables improved reconstruction of a transcriptome from RNA-seq reads. Nat Biotechnol 2015; 33 (3).

4 Simon A, Paul Theodor P, Wolfgang H. HTSeq--a Python framework to work with high-throughput sequencing data. Bioinformatics 2014; 31 (2).

5 Adam F, Mark D, Anne-Maud F et al. GENCODE reference annotation for the human and mouse genomes. Nucleic Acids Res 2018; 47 (0).

6 Yi Z, Hui L, Shuangsang F et al. NONCODE 2016: an informative and valuable data source of long non-coding RNAs. Nucleic Acids Res 2015; 44 (0).

7 Geng C, Ziyun W, Dongqing W et al. LncRNADisease: a database for long-non-coding RNA-associated diseases. Nucleic Acids Res 2012; 41 (0).

8 Junhao Z, Yali W, Liye T et al. Circ-RAPGEF5 promotes intrahepatic cholangiocarcinoma progression by stabilizing SAE1 to facilitate SUMOylation. J Exp Clin Cancer Res 2023; 42 (1).

9 Shi B XD, Yao Y. A water-soluble supramolecular polymer constructed by pillar [5] arene-based molecular recognition. Chemical Communications; 2014, 50(90): 13932-13935.

10 Zhang Z SL, Yang J. Assembly of a self‐complementary monomer: Formation of a pH‐responsive pillar [5] arene‐based supramolecular polymer. Journal of Polymer Science Part A: Polymer Chemistry; 2018, 56(3): 261-265.

11 Wang Q, Wang H, Yan H et al. Suppression of osteoclast multinucleation via a posttranscriptional regulation-based spatiotemporally selective delivery system. Science advances 2022; 8 (26): eabn3333.

**Supplementary documents**

**Figure S1. ECM stiffness could affect the malignant phenotype, intracellular mechanical conduction, and nuclear transcriptional activity of HCC cells.**

1. Cell experiments exhibited that ECM stiffness (16kPa vs 2kPa) could affect the malignant phenotype of HCC cells——cell morphology under optical microscopy (scale bar=50μm) (The time points for the assay were chosen at 48 hours after treatment);
2. The multicolor fluorescence images of DAPI/Phalloidin/YAP1 in HCC cells under different ECM stiffness conditions (16kPa vs 2kPa) (scale bar=100μm) (The time points for the assay were chosen at 48 hours after treatment);
3. The multicolor immunofluorescence iamges of DAPI/COL1A1/ACTIN/YAP1 in HCC tissues under different ECM stiffness conditions (scale bar=50μm);
4. The western blot assays showing that ECM stiffness (16kPa vs 2kPa) could affect the content and activity of YAP in HCC (The time points for the assay were chosen at 48 hours after treatment).

**
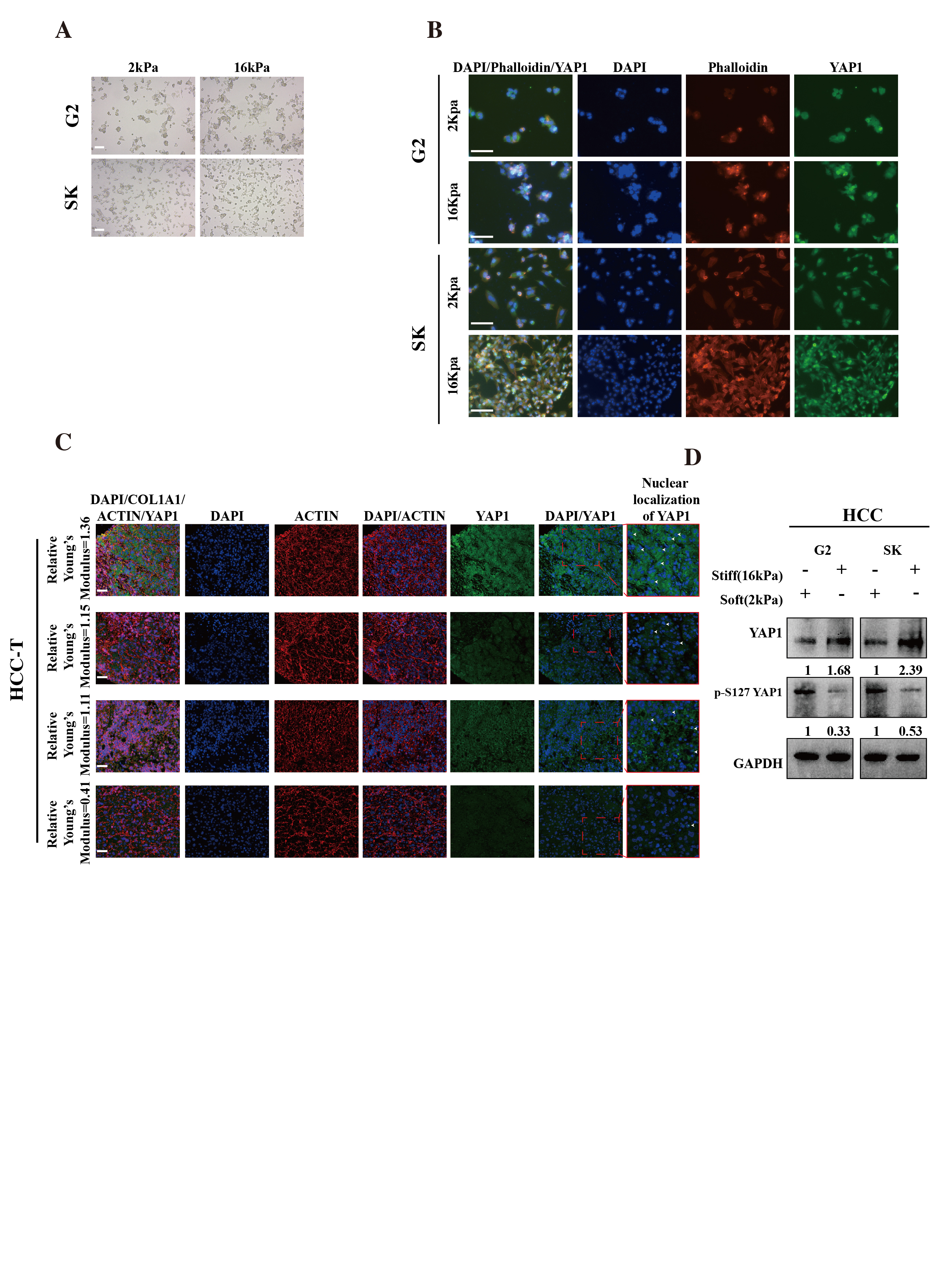
**

**Figure S2. The cell phenotype screening experiment performed with siRNA libraries containing proliferation and invasion assays.**

1. B) The presentation of phenotype screening results adopting siRNA library, including proliferation (The time points for the assay were chosen at 0, 1, 2, 3, and 4 days after treatment) and invasion assays (The time points for the assay were chosen at 48 hours after treatment) (Only the phenotypes screening results of the 15 siRNAs with the most obvious differences were shown) (scale bar=50μm) (n=3);
2. The knockdown efficiency of the top 15 siRNAs with the most significant phenotypic differences (The time points for the assay were chosen at 48 hours after treatment) (n=3).

The continuous variables of normal distribution were represented as the mean ± standard error of the mean. Student's t-test was employed to compare continuous variables following normal distribution (A, C). P value <0.05 was considered statistically significant. *p < 0.05, **p < 0.01, ***p < 0.001.

**
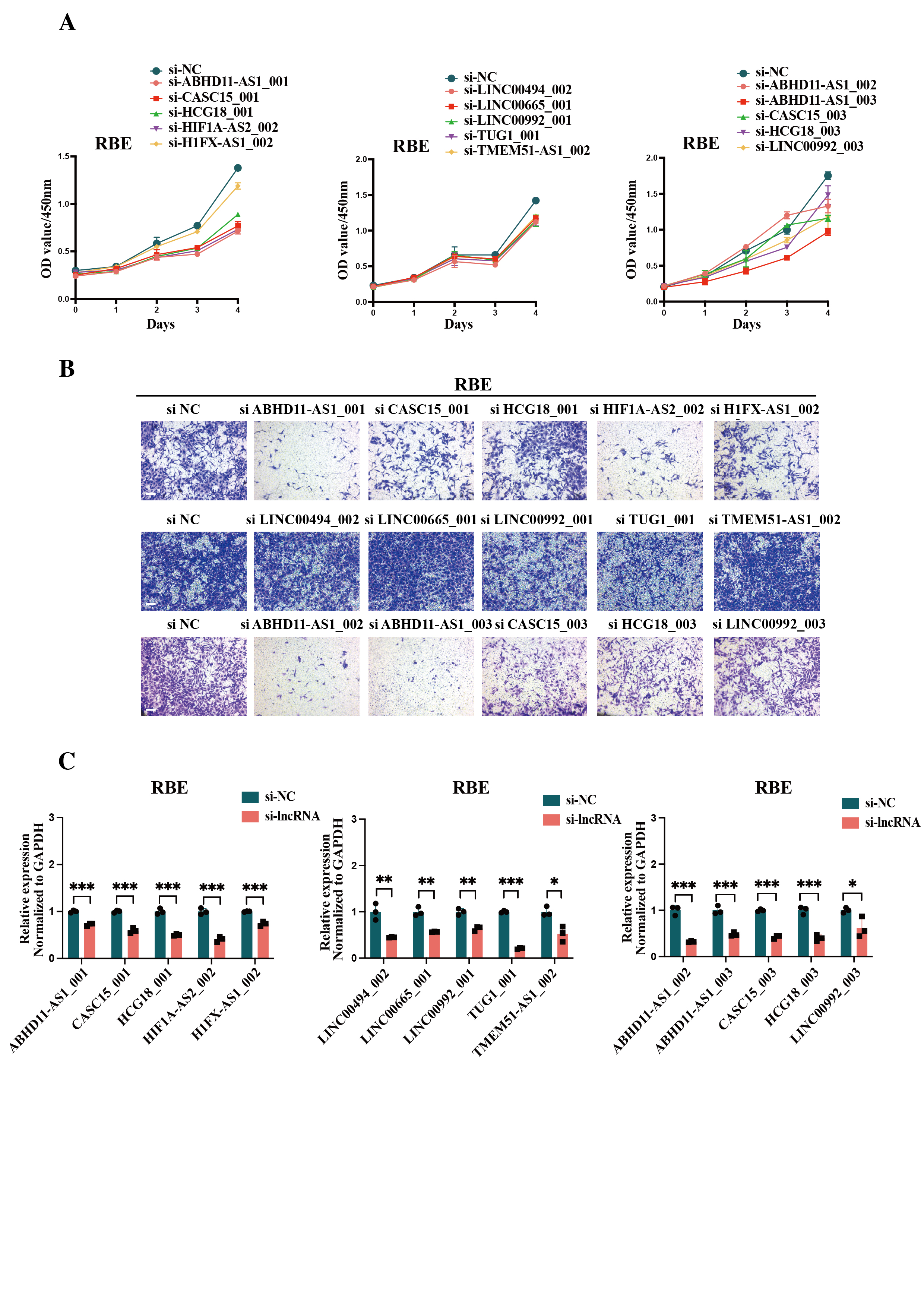
**

**Figure S3. Effects of ABHD11-AS1 on the proliferation and invasion in vitro.**

1. The volcano plot of differentially expressed lncRNAs between ICC and paired adjacent tissues according to the transcriptome sequencing of 19 patients and the location of ABHD11-AS1;
2. The impact of ABHD11-AS1 levels on long-term prognosis of ICC (based on TCGA-CHOL data);
3. The identification of new transcripts of ABHD11-AS1 in ICC cell lines (RBE and HuCCT1) through the RACE assay;
4. The qRT-PCR results of overexpressing different transcripts of ABHD11-AS1 in ICC cell lines (The time points for the assay were chosen at 48 hours after treatment) (n=3);
5. The expression levels of ABHD11-AS1 in normal biliary epithelial cell line HIBEpiC and ICC cell lines (RBE, HuCCT1, and 9810) (n=3);
6. G) Phenotype experiments exhibiting the impact of targeting ABHD11-AS1 with three different siRNAs on the proliferation (The time points for the assay were chosen at 0, 1, 2, 3, and 4 days after treatment) and invasion (transwell, scale bar=50μm) (The time points for the assay were chosen at 48 hours after treatment) abilities of ICC cell lines (RBE, HuCCT1, and 9810) (n=3);

H-I) Phenotype experiments exhibiting the impact of overexpressing different transcripts of ABHD11-AS1 on the proliferation (The time points for the assay were chosen at 0, 1, 2, 3, and 4 days after treatment) and invasion (transwell, scale bar=50μm) (The time points for the assay were chosen at 48 hours after treatment) abilities of ICC cell lines (RBE and HuCCT1) (n=3).

The continuous variables of normal distribution were represented as the mean ± standard error of the mean. The ANOVA test was employed for comparing data of multiple groups (D-E, G, I). Survival analysis was performed using the Kaplan-Meier method, and survival curves were simultaneously plotted; The log-rank test was used to compare the different groups' overall survival (B). P value <0.05 was considered statistically significant. *p < 0.05, **p < 0.01, ***p < 0.001.

**
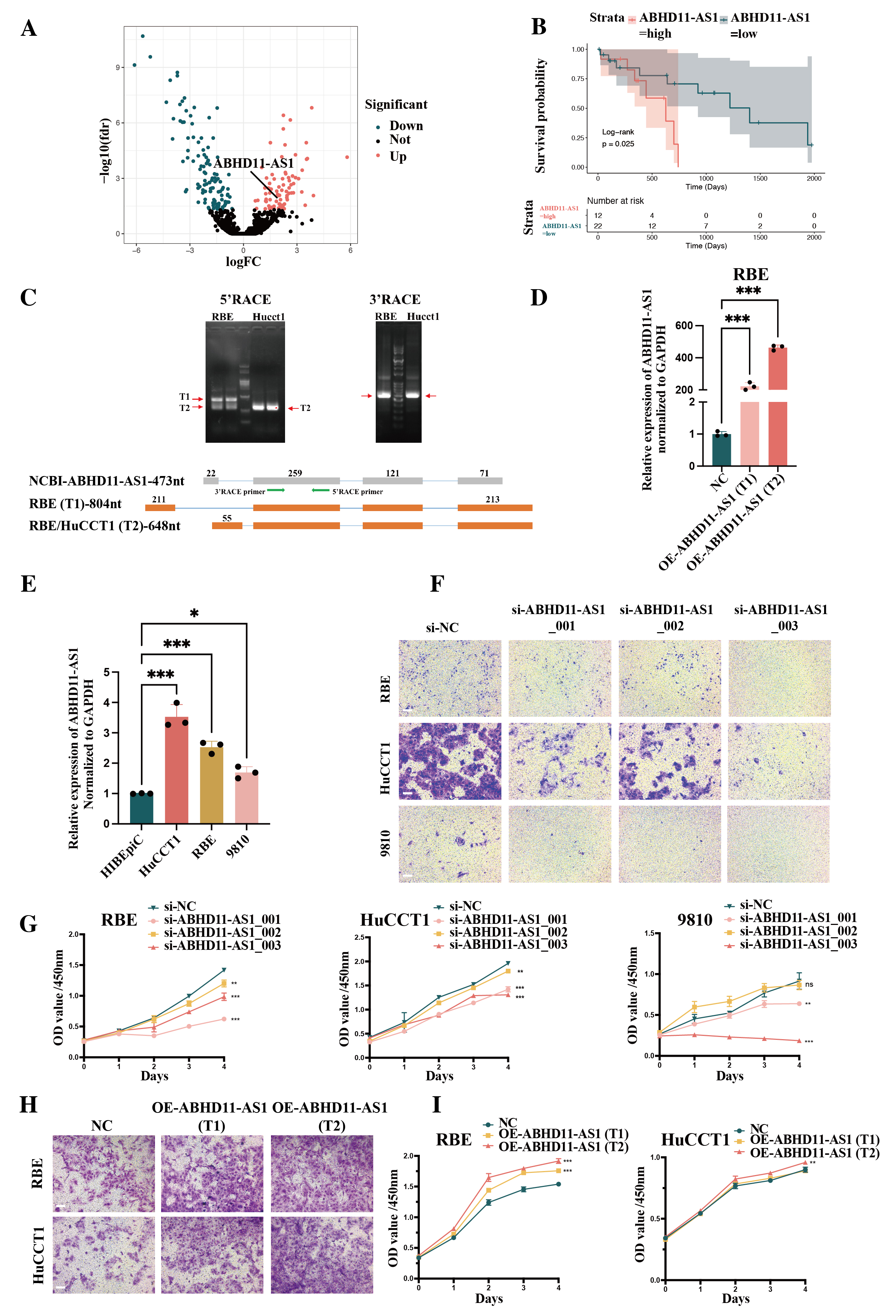
**

**Figure S4. The comparison of the composition of the extracted cell membrane (HIBEpiCM, RBEM, and HuCCT1M) according to the results of protein mass spectrometry analysis (Label-free quantification, LFQ).**

1. The results of the overlap between the differential proteins of HIBEpiCM vs RBEM (n=733) and the differential proteins of HIBEpiCM vs HuCCT1M (n=724) (low expression in HIBEpiCM) obtained by protein mass spectrometry analysis (Label-free quantification, LFQ) on the the extracted cell membrane (HIBEpiCM, RBEM, and HuCCT1M) (resulting in 318 overlapped proteins);
2. The Reactome enrichment analysis results of these 318 overlapped proteins;
3. A summary of proteins belonging to both the overlapped proteins and the INTEGRIN_CELL_SURFACE_INTERACTIONS pathway;
4. The validation of the mass spectrometry results using western blot.

**
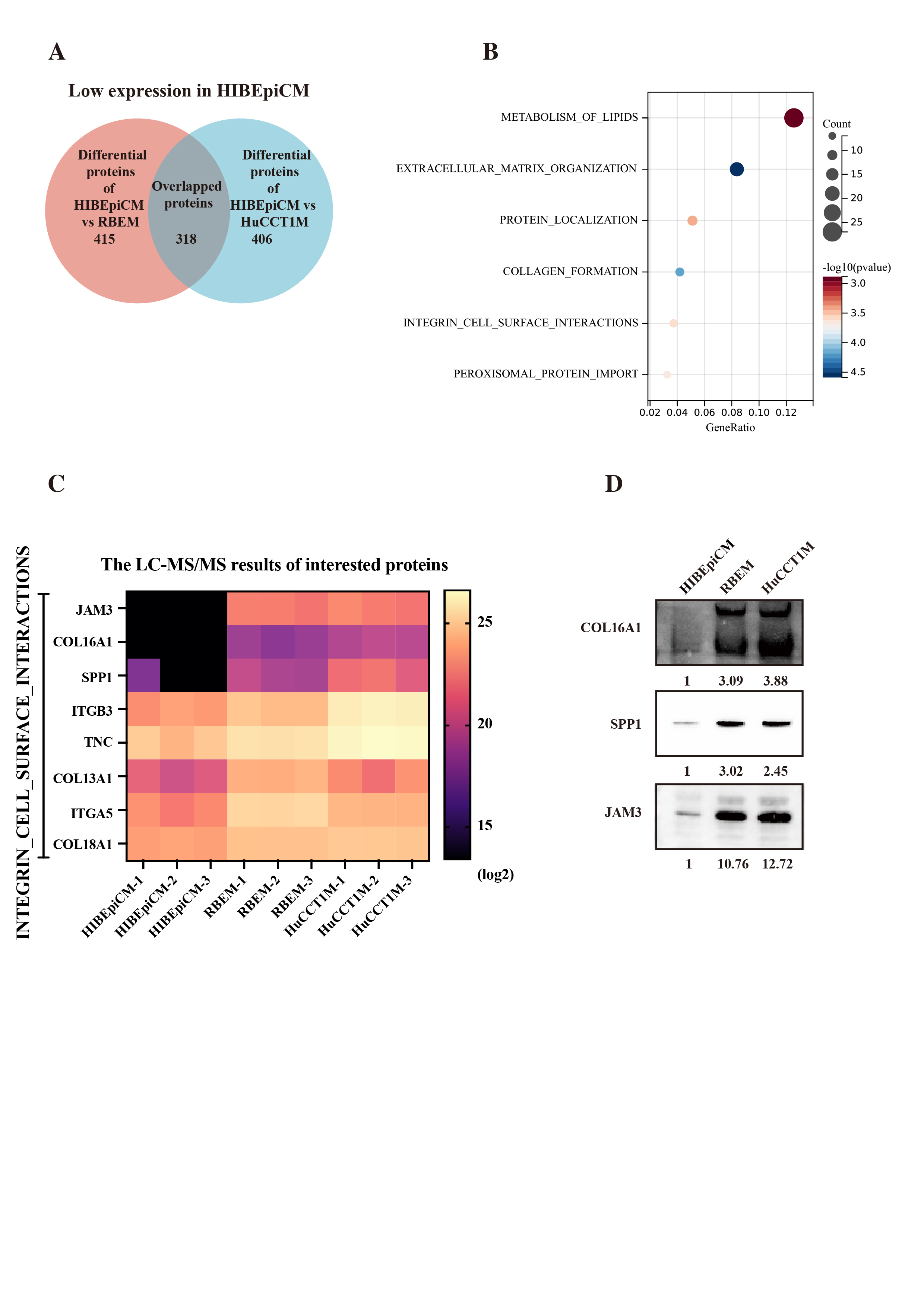
**

**Figure S5 The validation of the transfection efficiency of NPs+siRNA in vitro and the comparison of bodyweight change of five groups during the whole course of experimental timeline**

1. The synthetic route to CBP5;
2. The visualization of the transfection efficiency in RBE cells after coculture with lipo3000+siRNA-FAM or NPs+siRNA-FAM at the indicated N/P ratio for 48 hours (scale bar=100μm);

C-D) The results of qRT-PCR and western blot reflecting the transfection efficiency in RBE cells after coculture with lipo3000+siABHD11-AS1 or NPs+siABHD11-AS1 at the indicated N/P ratio=20 for 48 hours (n=3).

E) The comparison of bodyweight change of five groups during the whole course of experimental timeline (corresponding to Figure 8) (n=4);

The continuous variables of normal distribution were represented as the mean ± standard error of the mean. Student's t-test was employed to compare continuous variables following normal distribution (C). The ANOVA test was employed for comparing data of multiple groups (E). P value <0.05 was considered statistically significant. *p < 0.05, **p < 0.01, ***p < 0.001.

**
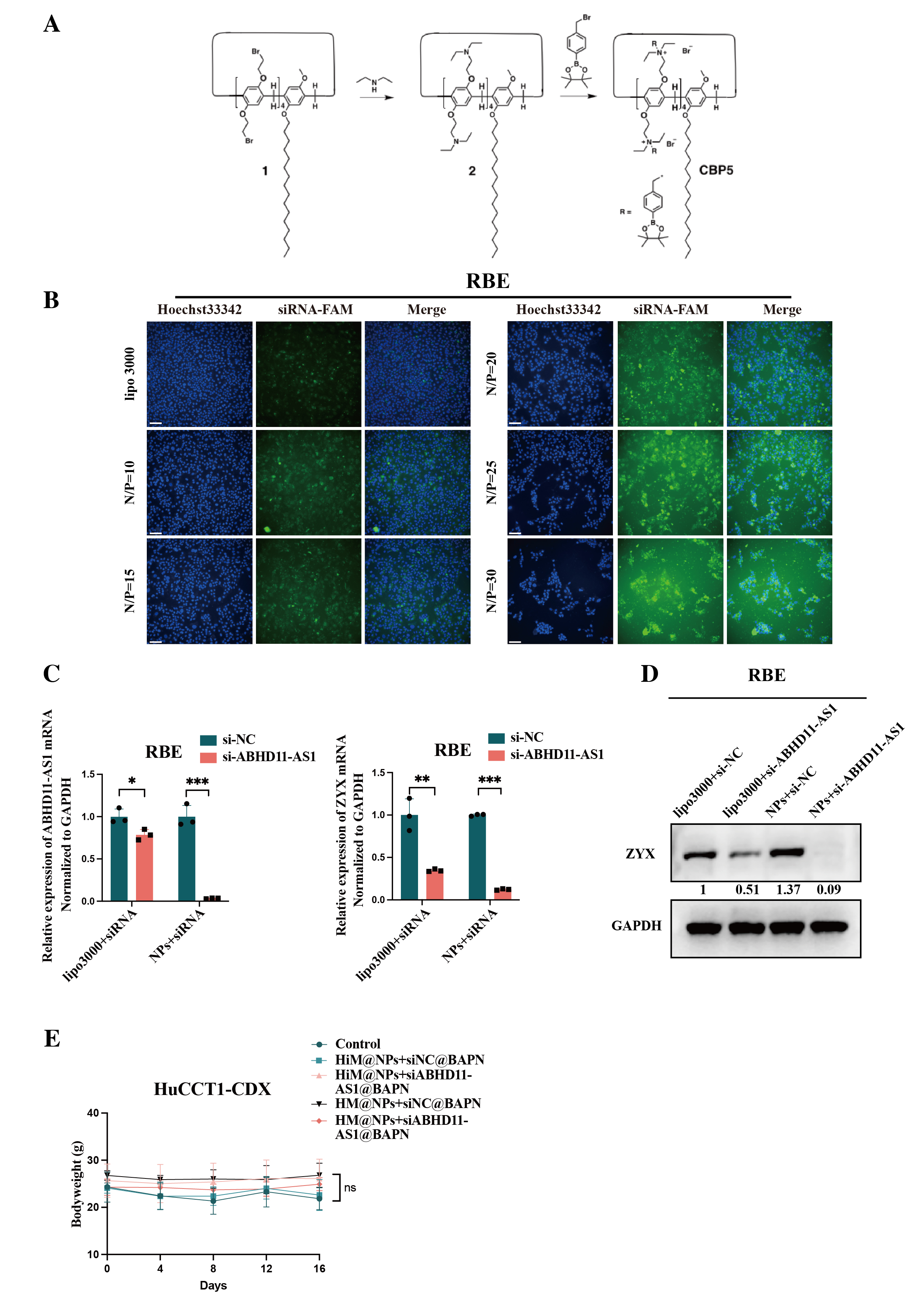
**

**Figure S6 The repeated experiment and statistical graph corresponding to Figure 4L.**

**
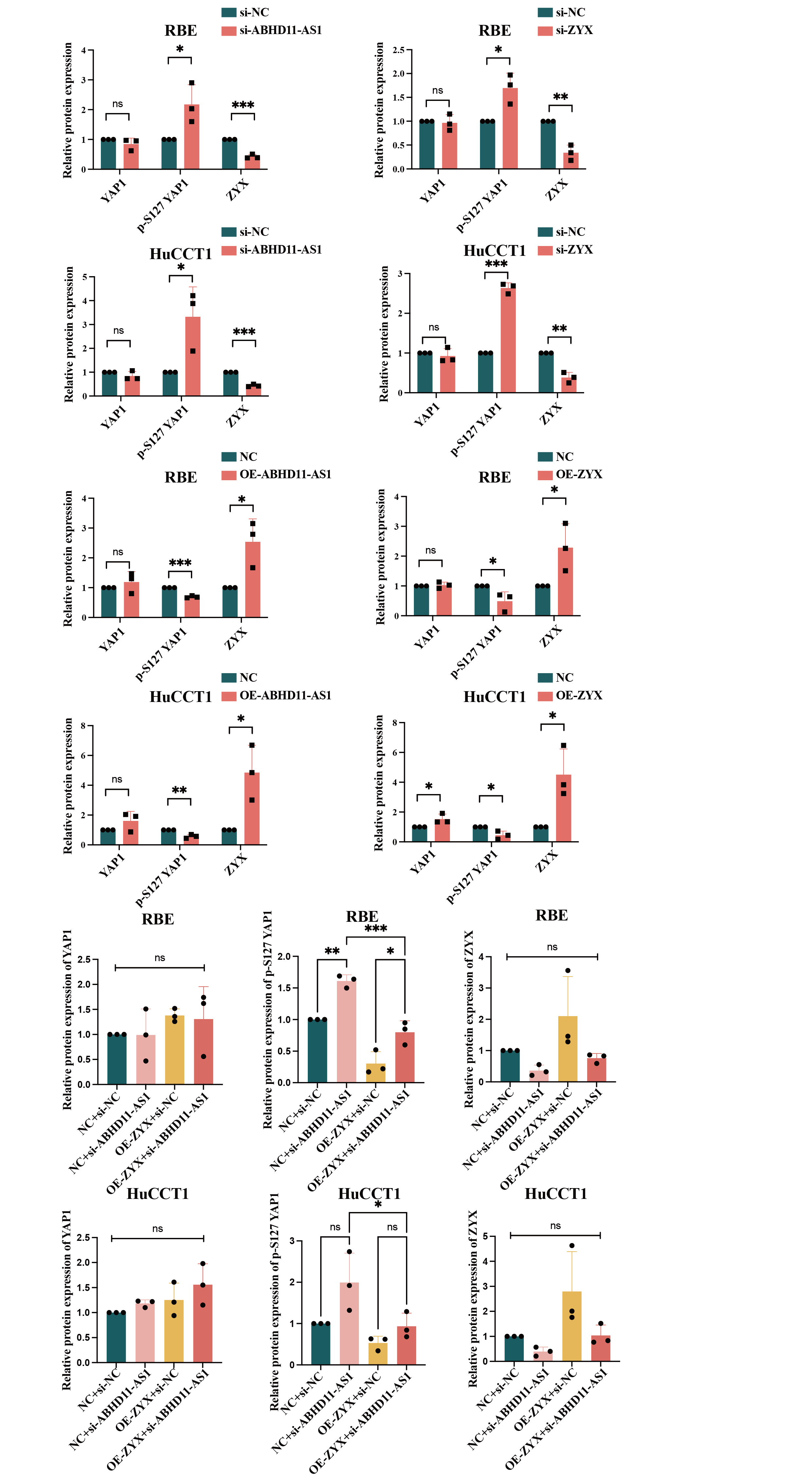
**

**
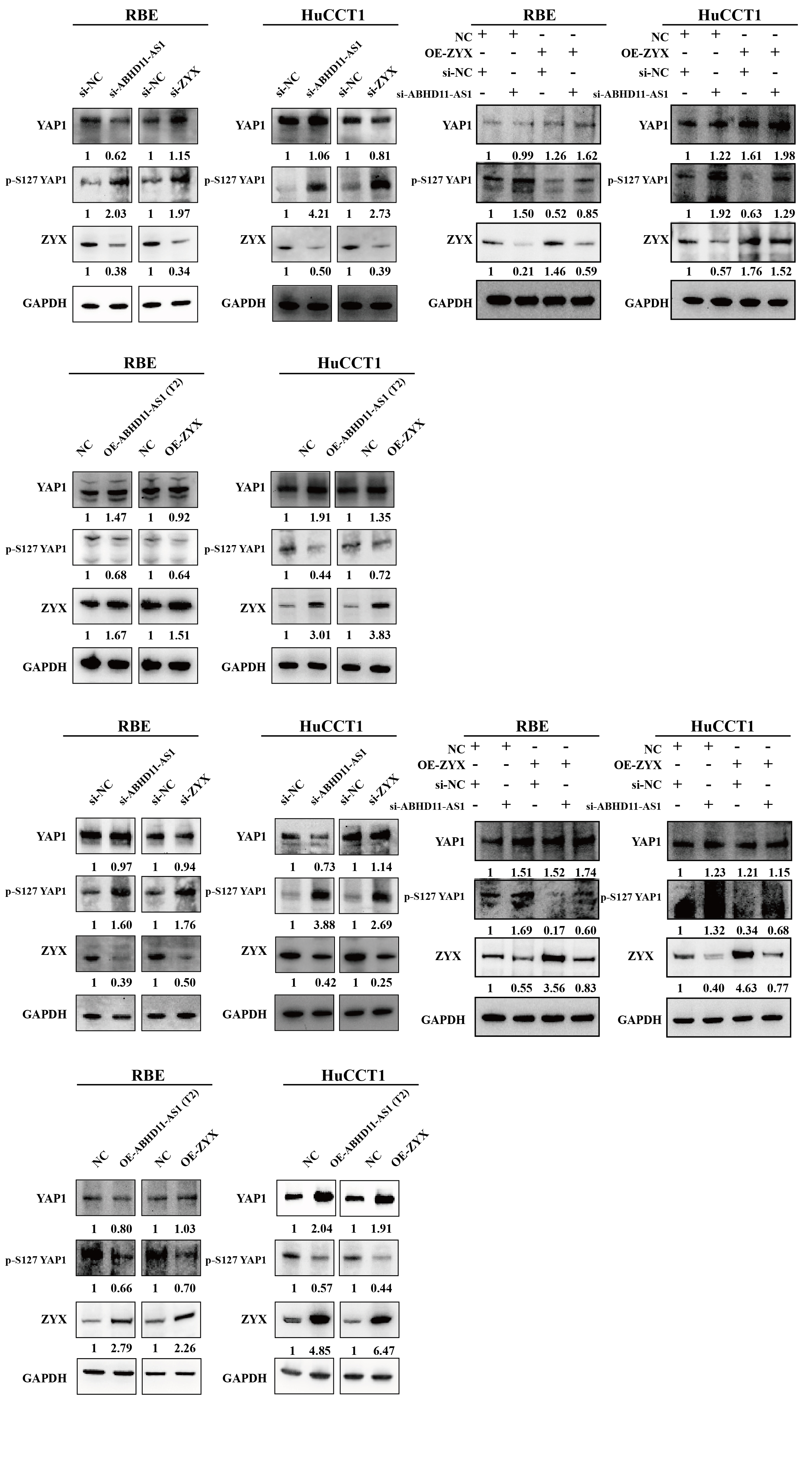
**

**Table S1. siRNA used in this study.**

| **Symbol** | **Target sequence** |
| --- | --- |
| si-ZYX | GGCAGAATGTGGCTGTCAA |
| si-U2AF1 | GTCGAGAGATCGTGAAAGA |
| si-STAU2 | GAAAGTGGACCACCACATA |
| si-FBL | GGGCTAAGGTTCTCTACCT |
| si-HTATIP2 | CCGGCAGAGTGCTCTTAAA |
| si-CAPNS1 | GGACCATTTGCAGTAGTGA |
| si-YAP1 | CCACCAAGCTAGATAAAGA |
| si-ABHD11-AS1_001 | GGACCAAGTCCTCCAGGAA |
| si-ABHD11-AS1_002 | CACCTGACAGCAACATCAA |
| si-ABHD11-AS1_003 | GAGACACCTCTTCCAGACA |
| si-TUG1_001 | GCACCTGGAACCTCATCTA |
| si-TUG1_002 | CCATCATGATGTGGCCTTT |
| si-TUG1_003 | CGACTTGATTACCAAAGAA |
| si-LINC00665_001 | GGTGCAAAGTGGGAAGTGT |
| si-LINC00665_002 | GGGTGGGAAATTGGCCATT |
| si-LINC00665_003 | CCGTATTGTCTCCAGATTA |
| si-CASC15_001 | CCCTCAGGTGACTACAGAT |
| si-CASC15_002 | GCTCAACCACATCTAATTT |
| si-CASC15_003 | GCAACATGCTTCACTGTCT |
| si-LINC00494_001 | CAGGATCACCAAGGTCTCT |
| si-LINC00494_002 | CTTCAACCATCTGGCTTCT |
| si-LINC00494_003 | CCACCTCCCTGCTGTTCCT |
| si-LINC00992_001 | GGTCAGCCAATGAGACAAT |
| si-LINC00992_002 | CCCTGAATGGCACTATAAT |
| si-LINC00992_003 | TCAGCCAATGAGACAATTT |
| si-TMEM51-AS1_001 | CGGAATTCATGGGTACATA |
| si-TMEM51-AS1_002 | GGAAGAAGAGAAACAAGAATT |
| si-TMEM51-AS1_003 | GCCATCCTTGTGCTCAGCA |
| si-HCG18_001 | AGCTGAAAGTCGACGAAGA |
| si-HCG18_002 | GCAAGTAAAGGAGATCATA |
| si-HCG18_003 | AAGCAAGAGATGTGGATTT |
| si-H1FX-AS1_001 | CCGCAAGAGGAGCTTTAAT |
| si-H1FX-AS1_002 | GGAATTCCTGTGAGAGGAT |
| si-H1FX-AS1_003 | GCTTTCCGTGGCTTTGCTA |
| si-HIF1A-AS2_001 | GGCACTTCCTACATAATTT |
| si-HIF1A-AS2_002 | GCTTCACTCATCCCATTCA |
| si-HIF1A-AS2_003 | GGTGATGGCACTAAGATAA |
| si-SPINT1-AS1_001 | GCAGCCAGACAGACGGACA |
| si-SPINT1-AS1_002 | CAGGATCGAAGAGGGCGCA |
| si-SPINT1-AS1_003 | CCGCTAGGGAGCTCAAGTA |
| si-CYTOR_001 | CAAATTGACATTCCAGACA |
| si-CYTOR_002 | GAAACAGGAAGCTCTATGA |
| si-CYTOR_003 | CACACTTGATCGAATATGA |
| si-SNHG1_001 | CCAGCATCTCATAATCTAT |
| si-SNHG1_002 | CCAGCACCTTCTCTCTAAA |
| si-SNHG1_003 | TCTAAAGCCCAAGAGGAGT |
| si-PVT1_001 | GTGACCTTGGCACATACAG |
| si-PVT1_002 | GAGCTGCGAGCAAAGATGT |
| si-PVT1_003 | GGCACCTTCCAGTGGATTT |
| si-LINC02595_001 | TGAGAGACATGTTTGTAAT |
| si-LINC02595_002 | GCAGCTGGAGACTGCAACA |
| si-LINC02595_003 | AGAAGTGGTTTCTGAAAGA |
| si-AFAP1-AS1_001 | GGTGGAGAATGAACATTCT |
| si-AFAP1-AS1_002 | CTCGTTGTGAAACTTAAAT |
| si-AFAP1-AS1_003 | GGGCTTCAATTTACAAGCA |
| si-UCA1_001 | CCATCAGATCCTTGCCCAT |
| si-UCA1_002 | GGTAATGTATCATCGGCTT |
| si-UCA1_003 | GCTAACTGGCACCTTGTTA |
| si-AGAP2-AS1_001 | CCACTTGTTACCTGCTTTA |
| si-AGAP2-AS1_002 | GACCAGGGATCAACGGAAA |
| si-AGAP2-AS1_003 | CCACCTCAAACTCTTACCT |
| si-LINC00511_001 | CCAAGTTAGCCTCTCCCTT |
| si-LINC00511_002 | CCATCGATCGACCTACAAA |
| si-LINC00511_003 | GCTTGTGCCCTTGGAATTA |
| si-GAS5_001 | GCAAGCCTAACTCAAGCCA |
| si-GAS5_002 | GGACCAGCTTAATGGTTCT |
| si-GAS5_003 | GCAAAGGACTCAGAATTCA |
| si-LINC00941_001 | CACTACACTCAGCCAAATA |
| si-LINC00941_002 | GGCATACTGACAATACAAA |
| si-LINC00941_003 | GAGATCACTTTCACAATGT |
| si-ZFAS1_001 | GAGGGGAGCGGACCGCGGG |
| si-ZFAS1_002 | GCGTTTCGGGTCCAGTGCG |
| si-ZFAS1_003 | AGAAACTGGCGATGGAATA |
| si-PWAR6_001 | CCATCTGTGAGTGATACAA |
| si-PWAR6_002 | CGTCAACTCCCATGATGAT |
| si-PWAR6_003 | CTCAATCTTACCAGTCATT |
| si-HOXB-AS3_001 | GGTCAGACTTCGCTTGAAA |
| si-HOXB-AS3_002 | GACATCAACAGTTTCCAGA |
| si-HOXB-AS3_003 | CAAGTAGACTTCGCTTGAA |
| si-PACERR_001 | TGCAGCACATACATACATA |
| si-PACERR_002 | GTAGCTAAGTTGCTTTCAA |
| si-PACERR_003 | CCACGGGTCACCAATATAA |
| si-LHFPL3-AS2_001 | CAGTCTTCAGCCCTACATT |
| si-LHFPL3-AS2_002 | GGAATAAGGTCAGCATTAA |
| si-LHFPL3-AS2_003 | GAAGGAAAGGTCACCCAAT |
| si-LINC02210_001 | GGTAAATTGTACAGTACAT |
| si-LINC02210_002 | GGAACCATCAAATTGTTCA |
| si-LINC02210_003 | GCAGCCCACTCCTGTTTAA |
| si-SNHG6_001 | GGTGCAAGAAAGCCTTTGA |
| si-SNHG6_002 | GCGGCATGTATTGAGCATA |
| si-SNHG6_003 | ACCGGCGAGGGAGGAAGAA |
| si-NRAV_001 | GGATGGATAGTTCAGAGTA |
| si-NRAV_002 | GCAACACAGAATAGACATT |
| si-NRAV_003 | CCACTAGATGCCAATCACA |
| si-MELTF-AS1_001 | GACCAGAAGTAGTATGAGA |
| si-MELTF-AS1_002 | AAGGACATCTGGAGAACAT |
| si-MELTF-AS1_003 | CTGCAAATTGCTTTACATA |
| si-LINC01133_001 | GGAGGTAAAGAGTAGAAGA |
| si-LINC01133_002 | GGAACCTCAAGTCTATGCA |
| si-LINC01133_003 | CAACATGACCGGGAAGATT |
| si-ITGB1-DT_001 | CAGCCAAACCCAATTCTTA |
| si-ITGB1-DT_002 | GATATACACTCATGAAGTA |
| si-ITGB1-DT_003 | GAGGCAGCTTCTCAAAGTA |
| si-LINC01833_001 | CTCCCTTTCCAAATGTTCT |
| si-LINC01833_002 | TATGAGCCACCCAGTTTAA |
| si-LINC01833_003 | TGTCTCTTGGGTTGAGAAA |
| si-LINC00342_001 | GTAAGATACTCCACAAGAA |
| si-LINC00342_002 | GGTCAACATTCTTAGAGAA |
| si-LINC00342_003 | GCCTGACTGTTCAAAGGAA |
| si-MNX1-AS1_001 | GAACAACGCAGACAACATA |
| si-MNX1-AS1_002 | CTGCCTGCATGCTTTACCA |
| si-MNX1-AS1_003 | GGTCGAACCTTATCTGCTA |
| si-TERC_001 | CCTTCCACCGTTCATTCTA |
| si-TERC_002 | ACCGTTCATTCTAGAGCAA |
| si-TERC_003 | GCGAAGAGTTGGGCTCTGT |
| si-LINC01559_001 | GTAGGTGACTACAGTTAAT |
| si-LINC01559_002 | GCAAGAAGCTGGAAATCGA |
| si-LINC01559_003 | GCCATGACCTTGAATAAGT |
| si-FBXL19-AS1_001 | GACATACACCTGACTAGTA |
| si-FBXL19-AS1_002 | CTTCAACTGCCTATGCAAA |
| si-FBXL19-AS1_003 | GTCTCAAACCAATCAATCA |
| si-LINC01614_001 | GGTCAAGGATGAAAGGAAA |
| si-LINC01614_002 | GGGCGATCTAGATTATCAA |
| si-LINC01614_003 | GCTATACAATGACAGGAAA |
| si-ARAP1-AS1_001 | CTCCCAAGTTCCTGACTTA |
| si-ARAP1-AS1_002 | ACCCGCTTTCTGCTGTTCT |
| si-ARAP1-AS1_003 | ACTTATGCCCATCTCTCCA |
| si-LINC02331_001 | CTGAGGGCTTTGAAACATA |
| si-LINC02331_002 | CTGGATACCAATGCTTCTA |
| si-LINC02331_003 | GCCAAGACTGAAATCACCA |
| si-SLC44A3-AS1_001 | TGTGCACTTTGACGACTAA |
| si-SLC44A3-AS1_002 | GTATTCCACATAGCAAACA |
| si-SLC44A3-AS1_003 | TGACAAGGCAACAATAGAA |
| si-SCARNA9_001 | GCGTATGTATGTCTATGAA |
| si-SCARNA9_002 | CTACTGATCTTTGTAACTA |
| si-SCARNA9_003 | TGATGGTTTCTACACTTGA |
| si-TBILA_001 | CCACATCCATCAACTTTCA |
| si-TBILA_002 | TAGCCGAAATACCCTGGAA |
| si-TBILA_003 | ACAGAATCCTGACCATCAT |
| si-LINC02086_001 | CAACCTTTCCCTTCTGTTT |
| si-LINC02086_002 | CTCTTTGTCCTCTGAAGTA |
| si-LINC02086_003 | CTACATCTCGGCAAATACA |
| si-LINC01836_001 | CCGGAATTCTCTACAATAT |
| si-LINC01836_002 | ATCTCATTCTTGGCTGTGA |
| si-LINC01836_003 | ATCTGGAAGTGACCAAGAA |
| si-LINC01123_001 | ATGCTATCCAGCCCATCTA |
| si-LINC01123_002 | GTATCCATCACCATCGTTT |
| si-LINC01123_003 | CTATCAAAGTTTGCAAGGT |
| si-LINC01106_001 | AGGTCTGGATCTGTGATGA |
| si-LINC01106_002 | CACAAACCTAGAAGCTGGA |
| si-LINC01106_003 | GTGATGAGATCGGGAAAGT |
| si-FAM201A_001 | TCCTCTTCTTGTTGGTAAA |
| si-FAM201A_002 | CAAACCCAAAGTTACCTAA |
| si-FAM201A_003 | CGACTTCATACCAATATCT |
| si-LINC02615_001 | GACCTCTTCTCACCTCAAA |
| si-LINC02615_002 | AGCCATCAATCCAGGACAT |
| si-LINC02615_003 | GTACTCTTTCACCTCTTCT |
| si-FAM66B_001 | CACAGATGAAGAAAGTCAA |
| si-FAM66B_002 | GGACTATTTGGTCAGTGAA |
| si-FAM66B_003 | GGTTTCTCCCGATGCGATA |
| si-ZNF528-AS1_001 | GAGAGAGTAAAGAGAAAGA |
| si-ZNF528-AS1_002 | CTACCAAAGTGCTCGGATT |
| si-ZNF528-AS1_003 | CTTGCTTGTCTCAGATGAA |
| si-FAM66A_001 | CCCAGTTCAAGAATCTTCA |
| si-FAM66A_002 | GGATTCTGGTGTAGGTGGA |
| si-FAM66A_003 | CAGCTAGATGTCTGCATGA |
| si-LINC02577_001 | GCTTGAGAATTGCTATCAA |
| si-LINC02577_002 | GGATATGCTATGTCTCAAA |
| si-LINC02577_003 | CCGGCAAGTTGCAATGTAA |
| si-SPRY4-AS1_001 | GGAGGAAGCTAGTAGAAGA |
| si-SPRY4-AS1_002 | GGAGTAGGCTGACCAGTTA |
| si-SPRY4-AS1_003 | CACTGAGGTTTAAGTGCTA |
| si-LINC02212_001 | GGAGCTAATTTCACACTTT |
| si-LINC02212_002 | TCCCTGAAACCATCGATGA |
| si-LINC02212_003 | CACACTGTTATGGGATGAA |
| si-TEX41_001 | CCATTCATTTCCTCATTAG |
| si-TEX41_002 | GATCCAAACTCACACCATA |
| si-TEX41_003 | AGAGACAACACCAACATTT |
| si-LINC01287_001 | GCAGATACTGCTCTCATAA |
| si-LINC01287_002 | GGACAATGCAATCAACAAA |
| si-LINC01287_003 | TGAAGACACTCGACCATCT |
| si-KRT7-AS_001 | GAAACTCAACCACAGCTTT |
| si-KRT7-AS_002 | CCAACAATATGCCCATGAA |
| si-KRT7-AS_003 | GAATCCTAAGGGTTAAACA |
| si-HMGA2-AS1_001 | CGAAATAGCAGCACATGAT |
| si-HMGA2-AS1_002 | GCACATGATCCTCCTACCA |
| si-HMGA2-AS1_003 | AGGCACATGTTCCTATCTT |
| si-TPM1-AS_001 | CCGCAAGATTATAACCCAA |
| si-TPM1-AS_002 | CCTCAGGTTTGTCACAGAA |
| si-TPM1-AS_003 | CCTAATCCCAAAGGTAATA |
| si-LINC02154_001 | GTGTATTGGTTGAGGAACT |
| si-LINC02154_002 | GGTCAACAATCTCAACTCT |
| si-LINC02154_003 | CTCCCAATGTAACTGTCAA |
| si-SOX9-AS1_001 | CAAAGGAGCTAAAGAAGAA |
| si-SOX9-AS1_002 | GGCCATGGAGGCATGGTAT |
| si-SOX9-AS1_003 | AAGAAATCCACAATTTACC |
| si-THAP9-AS1_001 | AGCTACAGACAAGAATAGA |
| si-THAP9-AS1_002 | AGAGAGTTCTTCACCACAA |
| si-THAP9-AS1_003 | GCGCAATCCTGAAGACAAA |
| si-LINC01671_001 | TCAGAAATAACCCATCACA |
| si-LINC01671_002 | GGCAGACAAATCCTACAAA |
| si-LINC01671_003 | CACAGCAAAGAGCCAATTA |
| si-COL18A1-AS1_001 | TTAACAGCATGACAAGCCA |
| si-COL18A1-AS1_002 | CCAAGGTTGTGAGAAGATT |
| si-COL18A1-AS1_003 | AGCTCTGAGATCACAGTGT |
| si-LINC01956_001 | GCACAGTTTCGATTTGTTTAG |
| si-LINC01956_002 | GCTGAAGAGACCTGACTTTAT |
| si-LINC01956_003 | GCTTCAACTGGTCCTGAATGG |
| si-SNORA53_001 | GCTTCCTTAGATCCACCTTTG |
| si-SNORA53_002 | GGCATGTAAGAGAAATATACC |
| si-SNORA53_003 | GCATTTATGCTGCCATTTGTT |
| si-CCDC144NL-AS1_001 | GACCAATCTTCCTCTTCCT |
| si-CCDC144NL-AS1_002 | CTTTGAACAACACGGATTT |
| si-CCDC144NL-AS1_003 | CAACGTAGAGGACTAGGAA |
| si-LINC01480_001 | CGACCAATGGATTGACATA |
| si-LINC01480_002 | GGAGAAAGGCATAGCAGAA |
| si-LINC01480_003 | GCACATCAGTGCAAGAACA |
| si-SNORA74A_001 | GGTGCCTGTGATGGTGTTA |
| si-SNORA74A_002 | GATCCTAGTCTGGGTGCAA |
| si-SNORA74A_003 | GAGCAAACACTGTCTTTAT |
| si-LINC02298_001 | GGATGATATGGAAGAGTGA |
| si-LINC02298_002 | GCAAGAAGATCCTACTAGT |
| si-LINC02298_003 | GGTGCTTCCCAACCTTAGA |
| si-BCYRN1_001 | ACTTCCCTCAAAGCAACAA |
| si-BCYRN1_002 | GTAACTTCCCTCAAAGCAA |
| si-BCYRN1_003 | GCTCTCAGGGAGGCTAAGA |
| si-PCAT6_001 | TCCTTACTCTTGGACAACA |
| si-PCAT6_002 | GCTCATCTCTCCAATTCAA |
| si-PCAT6_003 | GTCCTACAACGTCTTGTTA |
| si-SNHG12_001 | GCAGTGTGCTACTGAACTT |
| si-SNHG12_002 | AGAGGTGATCAATAAAAGA |
| si-SNHG12_003 | GGTGATCAATAAAAGAAGA |
| si-LOXL1-AS1_001 | CCTTGCTCTTTCTGTTCTA |
| si-LOXL1-AS1_002 | CCTCCTAAATGGTCTATTT |
| si-LOXL1-AS1_003 | GACTGACTTATGTGGACAA |
| si-ADAMTS9-AS1_001 | GCAAAGCTAATCTGCTCAA |
| si-ADAMTS9-AS1_002 | GCAGTTTCCTTGTCACTTA |
| si-ADAMTS9-AS1_003 | CCATACTGATACAGCCAAA |
| si-DUBR_001 | GCTCCCAAAGTTGCAGACA |
| si-DUBR_002 | AGAGCATCCTCATCCATTT |
| si-DUBR_003 | TCTGACCAGCCTAGGCAAT |
| si-IGFL2-AS1_001 | CACATAACACCGTGTTTAA |
| si-IGFL2-AS1_002 | TGCAGAGTTCCTGATTTCA |
| si-IGFL2-AS1_003 | CCATGAGATCCACCCATGA |

**Table S2. Sequence of RT-qPCR primers and PCR primers.**

| **Symbol** | **Primer sequence** |
| --- | --- |
| ABHD11-AS1-F | GCAGGTACAGAGCACCTCAG |
| ABHD11-AS1-R | AGCGACCAAGTCTTGTCTGG |
| CASC15-F | GAAAGGAGTCCCCTACCTGCC |
| CASC15-R | AGGGGCTCTGGAAAATTAGACAGTG |
| HCG18-F | AGCTGAAAGTCGACGAAGAG |
| HCG18-R | GAAGTGACGTGCCAGCTTAG |
| HIF1A-AS2-F | GCCATCTATTACTTTTAAAGCTTGGG |
| HIF1A-AS2-R | GGGATGAGTGAAGCAGTTCTCAG |
| H1FX-AS1-F | GATGGGGAAGGGATTCGCTC |
| H1FX-AS1-R | TCTCCTTTGCTGTGTTCCCG |
| LINC00494-F | CAGAAAGGCTGTTGTCCATTCTTCTAG |
| LINC00494-R | GTCTTTATAGCTTGTAGGAGTGGAGGC |
| LINC00665-F | CCAGGTGCAAAGTGGGAAGT |
| LINC00665-R | GACGCAAAAGGCCAGGACTC |
| LINC00992-F | GAGAGCTTGATTAATAGTTGGTCAGCC |
| LINC00992-R | GAGGTCTGATGCAAAATGGTGATTC |
| TUG1-F | TACTCCTTGACCAGCAACTTTTTTG |
| TUG1-R | GTATTCCTTCCAGACCCGATAGATG |
| TMEM51-AS1-F | CCCCTTCCTATCTTCCCCAGGG |
| TMEM51-AS1-R | AGAATTGGAGACATTTCCCAGACC |
| GAPDH-F | GACAGTCAGCCGCATCTTCT |
| GAPDH-R | GCGCCCAATACGACCAAATC |
| ZYX_F | TTCCACATCGCCTGCTTCACCT |
| ZYX_R | CGCAGGTGTTACACTTCTCCAG |
| YAP1-F | TGTCCCAGATGAACGTCACAGC |
| YAP1-R | TGGTGGCTGTTTCACTGGAGCA |
| U2AF1-F | GAACGTCTGTGACAACCTGGGA |
| U2AF1-R | AGGCTTCTCTGAAGTCCGTCAC |
| STAU2-F | GAGTGGTCCAAAGCCTGGGTTT |
| STAU2-R | CCAGAGATTACTTTGTGGCGGC |
| FBL-F | GAGGCTTCCATTCTGGTGGCAA |
| FBL-R | CAGGTTCTTGGTGACCAGTGCA |
| HTATIP2-F | GCCTGTTTTCCAAAGTCACGCTC |
| HTATIP2-R | CCTTGAAAGGCAGAGGCGTAGT |
| CAPNS1_F | CAGTTCGACACTGACCGATCAG |
| CAPNS1_R | CCCACTTTCATCTGAGTAGCGTC |
| ZYX promotor region (-2000～-1081) -F | GGCTTGAGCGTGTCAGAGAG |
| ZYX promotor region (-2000～-1081) -R | AATCACTTTTGCAAACCCCACTG |
| ZYX promotor region (-1800～-1601) -F | CTGGCAACCCAGAAAGGGAC |
| ZYX promotor region (-1800～-1601) -R | TAGCTGTATGCGCTATATGACTCAG |
| ZYX promotor region (-1600～-1401) -F | TGAAGAGGCGTCAGTTTGGG |
| ZYX promotor region (-1600～-1401) -R | GTTGCCTTGCACCCCTGATA |
| ZYX promotor region (-1400～-1201) -F | GGTGCTCCTTGCCAGTAGAG |
| ZYX promotor region (-1400～-1201) -R | ACCCGTAGGCGCTTCATTTT |
| ZYX promotor region (-1200～-1001) -F | GGGGAAGTTTCCCAGTGCAA |
| ZYX promotor region (-1200～-1001) -R | AGACTGGATTTCTCAGCCTGC |
| ZYX promotor region (-1000～-801) -F | CTCCACCAAGCGCAGTCTC |
| ZYX promotor region (-1000～-801) -R | GCTCGTCTTTTACGGCCCTT |
| ZYX promotor region (-800～-601) -F | GAACGGTCCCTCTCGCAAC |
| ZYX promotor region (-800～-601) -R | TGGGGATACGAACTTCCAACG |
| ZYX promotor region (-600～-401) -F | CCAGCTCCATACGTTCGGC |
| ZYX promotor region (-600～-401) -R | CGAGGTAGCCACGCAGC |
| ZYX promotor region (-400～-201) -F | GGGGGACGGTGAGTCAGAT |
| ZYX promotor region (-400～-201) -R | CCGCCGAGGTCCGCT |
| ZYX promotor region (-200～-1) -F | CCTTCCTGGGGGAGTTTCCT |
| ZYX promotor region (-200～-1) -R | GTCCGGAGCAGCCTCCA |
| ABHD11-AS1 (T2) promotor region (-2000～-1081) -F | GATGTTAGGAGGGCGGGGT |
| ABHD11-AS1 (T2) promotor region (-2000～-1081) -R | ACCACCATGCCCAGCAAAT |
| ABHD11-AS1 (T2) promotor region (-1800～-1601) -F | ATTGAGCCCAGGAGTTTGAGG |
| ABHD11-AS1 (T2) promotor region (-1800～-1601) -R | GGATTACAAGTGTGACCCACATG |
| ABHD11-AS1 (T2) promotor region (-1600～-1401) -F | CCCGGATGTGGTGGCAT |
| ABHD11-AS1 (T2) promotor region (-1600～-1401) -R | ATGGACTCTTGCTCTGTCACCC |
| ABHD11-AS1 (T2) promotor region (-1400～-1201) -F | CCAGCACTTTGGGAGGCC |
| ABHD11-AS1 (T2) promotor region (-1400～-1201) -R | GGACTACAGGCGCCCGC |
| ABHD11-AS1 (T2) promotor region (-1200～-1001) -F | GCTTTAACCTGGGAGGCAGAG |
| ABHD11-AS1 (T2) promotor region (-1200～-1001) -R | CGAGTAGCCAGTGGCATGAG |
| ABHD11-AS1 (T2) promotor region (-1000～-801) -F | GAGCCAAGACTGCACCACTG |
| ABHD11-AS1 (T2) promotor region (-1000～-801) -R | GATTACAAGCGTGAGCCACC |
| ABHD11-AS1 (T2) promotor region (-800～-601) -F | TGAAACCCCGTCTCTACTGAAAGTA |
| ABHD11-AS1 (T2) promotor region (-800～-601) -R | GGTGCAATCTCAGCTCACTGC |
| ABHD11-AS1 (T2) promotor region (-600～-401) -F | TGAGGAGGAATTTGTTAGGGATACA |
| ABHD11-AS1 (T2) promotor region (-600～-401) -R | ATAAGGTACCCCAGCCCCC |
| ABHD11-AS1 (T2) promotor region (-400～-201) -F | GTGGTGTGATCCTATGGCTCACT |
| ABHD11-AS1 (T2) promotor region (-400～-201) -R | AAGAGGGTGCTCTACCCACG |
| ABHD11-AS1 (T2) promotor region (-200～-1) -F | GGGAGGAAGGGGGCTGAG |
| ABHD11-AS1 (T2) promotor region (-200～-1) -R | AGACATGGCCCTTTGCCC |

**Table S3. Antibodies used in this study.**

| **Symbol** | **Source** | **Identifier** | **Usage** |
| --- | --- | --- | --- |
| COL1A1 | Boster | BA0325 | IF |
| LOX | Abcam | ab174316 | IF |
| YAP1 | SANTA CRUZ | sc-376830 | Multicolor IF |
|  | CST | #14074 | WB, IF, ChIP |
| ACTIN | Abcam | ab179467 | IF |
| p-S127 YAP1 | Abmart | T55743 | WB |
| ZYX | Abcam | ab109316 | WB, IF |
| STAU2 | Proteintech | 15998-1-AP | WB, RIP |
| Na-K-ATPase | Abcam | ab76020 | WB |

**Table S4. The sequence of different ABHD11-AS1 transcripts in ICC as determined by RACE assays**

| **Symbol** | **Sequence** |
| --- | --- |
| ABHD11-AS1-T1 (804 nt) | AGACTATGGGCACAGGTTAGCTGGCTGCCAGGCAAGCTAGGGACACTGTTCCAGCTTTCCCTTCTTCCCTGGAAAATCTGGCCCAAGACGGTCCGATGTGGCCTGGCAGAGGGAACAGCCACACCCTGTTCCAGTTGTCCCGGCTTCAACTTTGGGTGCCCTGCAGAATGGGCATTGATGGCGCCCCACTTCACAGAGGAAAAGTAAGGAGGTCCATGAGACTGGGGCAGCAGCAGGGGCAGGTACAGAGCACCTCAGCCGCCTCTCCACCTGACAGCAACATCAAAGCCGAGGCCAAGGCGGGAGGGCCCAGAGTGAGACACCTCTTCCAGACAAGACTTGGTCGCTGCCTCCTCCTCTGTCACCCTGCGACACAGCCCAGAGTACCAGGCGGCTGGAGCACTGGGGGACACCCGGACAGAACCCTCCCTGGACCAAGTCCTCCAGGAACGGGATGAAGCCATTGCCAAGAAGCAGGCGGTGGAGGCGGAGCTGCAGAGATGCAAAGCCAGGCTACACGCCATGGAGGCCCAGCTGCTGGAGGTCCTGGAGGAGAAACTGAGGCTGAGGCGGGAGCTGGAGGCCTGGGAGGAGGACGTGCAGCAGCTGGTGTGGCAGCAGGTCCAGAATCAGCTGCAGAGAGAGGCCAAGGGTACTCGGGGAGCCCACGTGGACCCTGGAGCTGCCAGCACCCCCCGATCCAGATTCTCCCTGGGTCGGGGACGTTGGTGGTGACACAGACCTCAGCCCAGGACTTTGGGAGTAGTGTCTTTATTCATTAAAGCCTGAGGTCTGACCACCCCT |
| ABHD11-AS1-T2 (648 nt) | CTTGAGGGTAAGTGTTCTTCTGAACACAGCTGCCTCGTCCTTCCCTGATGACTGGGTCCATGAGACTGGGGCAGCAGCAGGGGCAGGTACAGAGCACCTCAGCCGCCTCTCCACCTGACAGCAACATCAAAGCCGAGGCCAAGGCGGGAGGGCCCAGAGTGAGACACCTCTTCCAGACAAGACTTGGTCGCTGCCTCCTCCTCTGTCACCCTGCGACACAGCCCAGAGTACCAGGCGGCTGGAGCACTGGGGGACACCCGGACAGAACCCTCCCTGGACCAAGTCCTCCAGGAACGGGATGAAGCCATTGCCAAGAAGCAGGCGGTGGAGGCGGAGCTGCAGAGATGCAAAGCCAGGCTACACGCCATGGAGGCCCAGCTGCTGGAGGTCCTGGAGGAGAAACTGAGGCTGAGGCGGGAGCTGGAGGCCTGGGAGGAGGACGTGCAGCAGCTGGTGTGGCAGCAGGTCCAGAATCAGCTGCAGAGAGAGGCCAAGGGTACTCGGGGAGCCCACGTGGACCCTGGAGCTGCCAGCACCCCCCGATCCAGATTCTCCCTGGGTCGGGGACGTTGGTGGTGACACAGACCTCAGCCCAGGACTTTGGGAGTAGTGTCTTTATTCATTAAAGCCTGAGGTCTGACCACCCCT |
